# Naphthalene diimides with improved solubility for visible light photoredox catalysis

**DOI:** 10.3762/bjoc.15.201

**Published:** 2019-08-27

**Authors:** Barbara Reiß, Hans-Achim Wagenknecht

**Affiliations:** 1 Institute of Organic Chemistry, Karlsruhe Institute of Technology (KIT), Fritz-Haber-Weg 6, 76131 Karlsruhe, Germany

**Keywords:** chromophore, dyes, electrochemistry, photochemistry, photoredox catalysis

## Abstract

Five core-substituted naphthalene diimides bearing two dialkylamino groups were synthesized as potential visible light photoredox catalysts and characterized by methods of optical spectroscopy and electrochemistry in comparison with one unsubstituted naphthalene diimide as reference. The core-substituted naphthalene diimides differ by the alkyl groups at the imide nitrogens and at the nitrogens of the two substituents at the core in order to enhance their solubility in DMF and thereby enhance their photoredox catalytic potential. The 1-ethylpropyl group as rather short and branched alkyl substituent at the imide nitrogen and the *n*-propyl group as short and unbranched one at the core amines yielded the best solubilities. The electron-donating diaminoalkyl substituents together with the electron-deficient aromatic core of the naphthalene diimides increase the charge-transfer character of their photoexcited states and thus shift their absorption into the visible light (500–650 nm). The excited state reduction potential was estimated to be approximately +1.0 V (vs SCE) which is sufficient to photocatalyze typical organic reactions. The photoredox catalytic activity in the visible light range was tested by the α-alkylation of 1-octanal as benchmark reaction. Irradiations were performed with LEDs in the visible light range between 520 nm and 640 nm. The irradiation by visible light together with the use of an organic dye instead of a transition metal complex as photoredox catalyst improve the sustainability and make photoredox catalysis “greener”.

## Introduction

Photocatalysis couples the physical process of light absorption to an organic-chemical reaction by means of time, space and energetics. In order to apply visible light for photocatalysis despite its rather low energy this coupling requires to be mediated by a sensitizing species – a photocatalyst. If the interacting mode between the sensitizer and the reactant is via charge transfer, it is named photoredox catalysis. This research field has been established over the past decade [1–20]. In principle, it is a sustainable method for catalysis because sunlight is an essentially unlimited and thereby “green” natural light source and LEDs – conveniently used for irradiation experiments in the laboratory – are cheap and energy-saving artificial sources for irradiations. The current “working horse” for photoredox catalysis is mainly [Ru(bpy)_3_]^2+^ [21], due to its strong MLCT (metal-to-ligand charge transfer) absorption, the excellent yield of its triplet state and the long lifetime, the versatile redox behavior (Ru^3+^ vs Ru^+^) in quenching processes and the chemical and photochemical robustness. Despite their positive photoredox catalytic behavior, transition metal complexes have disadvantages, including high costs due to limited availability, toxicity [22–23] and polluting properties [24]. This thwarts the principally “green” concept of photoredox catalysis. In order to avoid transition metals and enhance the sustainability further, organic compounds, mainly eosin Y [25], rhodamine 6G [26], 9-mesityl-10-methylacridinium perchlorate [27], 1,2,3,5-tetrakis(carbazol-9-yl)-4,6-dicyanobenzenes [28] and *N*-phenylphenothiazines [29] were applied as important alternative photoredox catalysts [30–31]. These studies conclusively showed that there is no universal photoredox catalyst for different organic transformations. Instead, each photoredox catalyst has its own reactivity profile and scope. In order to apply organic dyes in advanced photoredox catalysts in a versatile way, it is crucial that modifications can be easily incorporated into the core structure in order to tune optical and redox properties. Naphthalene diimides (NDIs) as the smallest possible rylene dyes are such an important class of organic dyes. In contrast to their bigger homologs perylene diimides which were rarely used for photoredox catalysis [32–34], NDIs have a lower tendency to self-aggregate due to their smaller aromatic surface and thus are slightly better soluble in organic solvents [35–38]. NDIs are intensively applied as functional dyes [39–40], for artificial photosynthesis [41–42], for molecular architectures by self-assembly [43–44], as molecular sensors [45–47] and for organic electronics [48–49], but yet nearly completely unexplored for photoredox catalysis. Core-unsubstituted NDIs are colorless compounds with high extinction coefficients at the border between UV-A and visible light. Their fluorescence quantum yields are rather low and fluorescence lifetimes are rather short due to ISC into the triplet state [50–51]. NDIs are reversibly reducible and their stable radical anions absorb in the visible to NIR light range [37]. The aromatic core of NDIs can be easily modified by substituents in order to tune their optical and redox properties as mentioned above [39–40,52]. The common synthetic approach for core-substituted NDIs (cNDIs) makes tailor-made dyes rather easily accessible. With respect to these unique properties, NDIs should also be explored for photoredox catalysis. We present herein the synthesis and characterization of NDI **1** as unsubstituted chromophore reference and cNDIs **2**–**6** as potential visible light photoredox catalysts. The cNDIs **2**–**6** differ by the alkyl groups at the imide nitrogens and at the nitrogens of the two core substituents at the core in order to enhance their solubility. In general, NDIs are electron-poor chromophores. The diamino substituents of cNDIs **2**–**6** are expected to increase the charge-transfer character of their photoexcited states in order to shift their absorption into the visible light and to improve their photoredox catalytic power. The photoredox catalytic activity was representatively tested by the MacMillan benchmark reaction [53]. For this type of photoredox catalytic reaction, solubility of cNDIs in DMF is a crucial prerequisite.

## Results and Discussion

### Synthesis of cNDIs **2**–**6** and their solubility

**Scheme 1:**
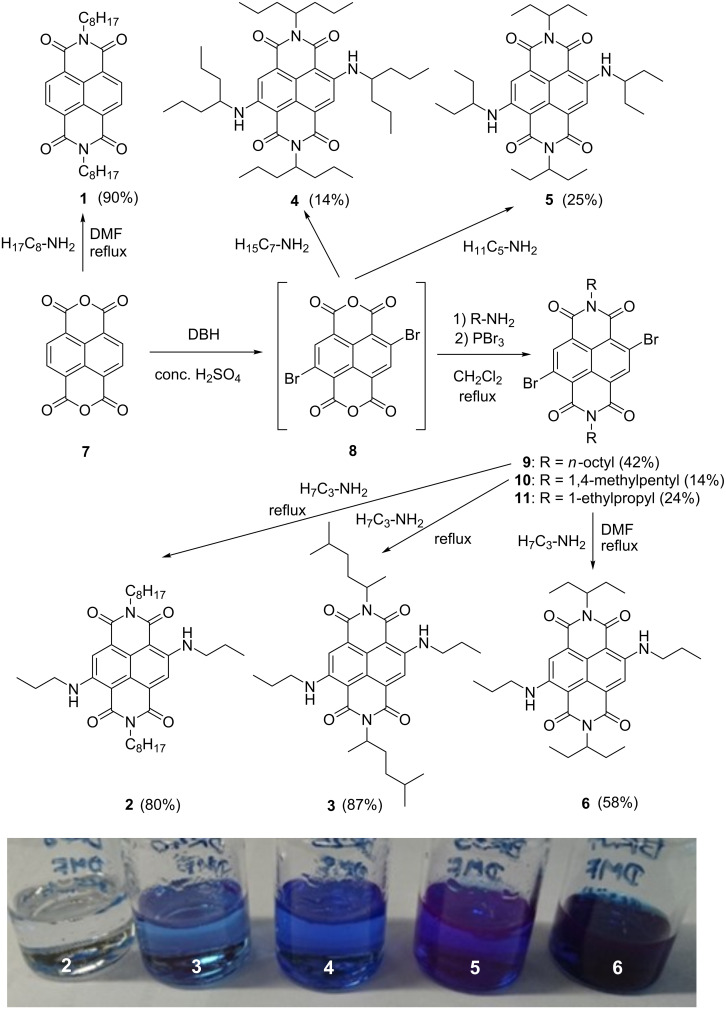
Synthesis of reference NDI **1** and cNDIs **2**–**6**; bottom: image of saturated solutions of cNDIs **2**–**6** in DMF.

The commercially available precursor for all NDIs is 1,4,5,8-naphthalenetetracarboxylic acid anhydride (**7**) and the synthesis of cNDIs follows a modular approach. Core-unsubstituted NDIs are typically prepared by condensation of **7** with 2–3 equivalents of the respective amine. The corresponding reaction of **7** with *n*-octylamine in DMF gave the reference NDI **1** in 90% yield (Scheme 1) [54]. The synthetic module for cNDIs with two substituents at the core is the 2,6-dibromo anhydride derivative **8** that can be synthesized from **7** by elementary bromine, dibromoisocyanuric acid, or 1,3-dibromo-5,5-dimethylhydantoine (DBH) in concentrated sulfuric acid, or oleum in good yields [55–57]. The regioselective introduction of just two substituents can be controlled by stoichiometry. Accordingly, the 2,6-dibromo derivative **8** was prepared by 1.5 equivalents DBH and further used as crude product because it cannot be purified due to its very poor solubility. The subsequent condensation with *n*-octylamine, 1,4-dimethylpentylamine and 1-ethylpropylamine in refluxing CH_2_Cl_2_ gave the cNDIs **9**–**11** in 42%, 14% and 24% yields, respectively. Finally, the two bromo groups in cNDIs **9**–**11** were substituted by *n*-propylamine as nucleophile in refluxing CH_2_Cl_2_ to the cNDIs **2**, **3** and **6** in 80%, 87% and 58% yields, respectively. The two cNDIs **4** and **5** carrying the same alkyl groups at the imide nitrogens and at the nitrogens of the core substituents were directly synthesized (in one step) by 1-propylbutylamine or 1-ethylpropylamine using pressure reaction vials. The yields were rather low, 14% for **4** and 25% for **5**, but they should be regarded with respect to the fact that the additional time-consuming isolations of the respective dibromo-cNDI intermediates were omitted. After purification by column chromatography, all five cNDIs **2**–**6** were resolved in benzene and lyophilized under reduced pressure.

In general, NDIs are well soluble in CH_2_Cl_2_. As mentioned in the introduction, solubility of cNDIs as potential photoredox catalysts in DMF or in mixtures of DMF with CH_2_Cl_2_ is a crucial prerequisite for their photoredox catalytic suitability. The solubility was determined by preparing saturated solutions of the respective NDI **1** or cNDIs **2**–**6** at 22 °C in pure DMF. The absorbance was measured after filtration of the solution and after redilution if the optical density in the cuvette with 1 cm path length exceeded 1. The reference NDI **1** with the two *n*-octyl substituents at the imide nitrogens showed a maximum concentration (*c*_max_) of 2.5 mM. Saturated samples in DMF gave the first impression of cNDIs **2**–**6** (see bottom image in Scheme 1), and merely by visible inspection, the solubility of the cNDIs follows the order from **2** to **6**. In fact, *c*_max_ of **2** is far below 1 mM and difficult to determine by this simple method. At the far end, the best and measurable solubilities showed cNDIs **5** and **6** with *c*_max_ of 0.6 mM and 2.7 mM, respectively. The 1-ethylpropyl groups as rather short and branched alkyl substituents at the imide nitrogens and the *n*-propyl groups as short and unbranched ones at the core amines gave the best combination to improve the solubility. This qualifies cNDI **6** as the best soluble potential visible light photoredox catalyst.

### Characterization of NDI **1**, cNDI **2** and cNDI **6**

**Figure 1:**
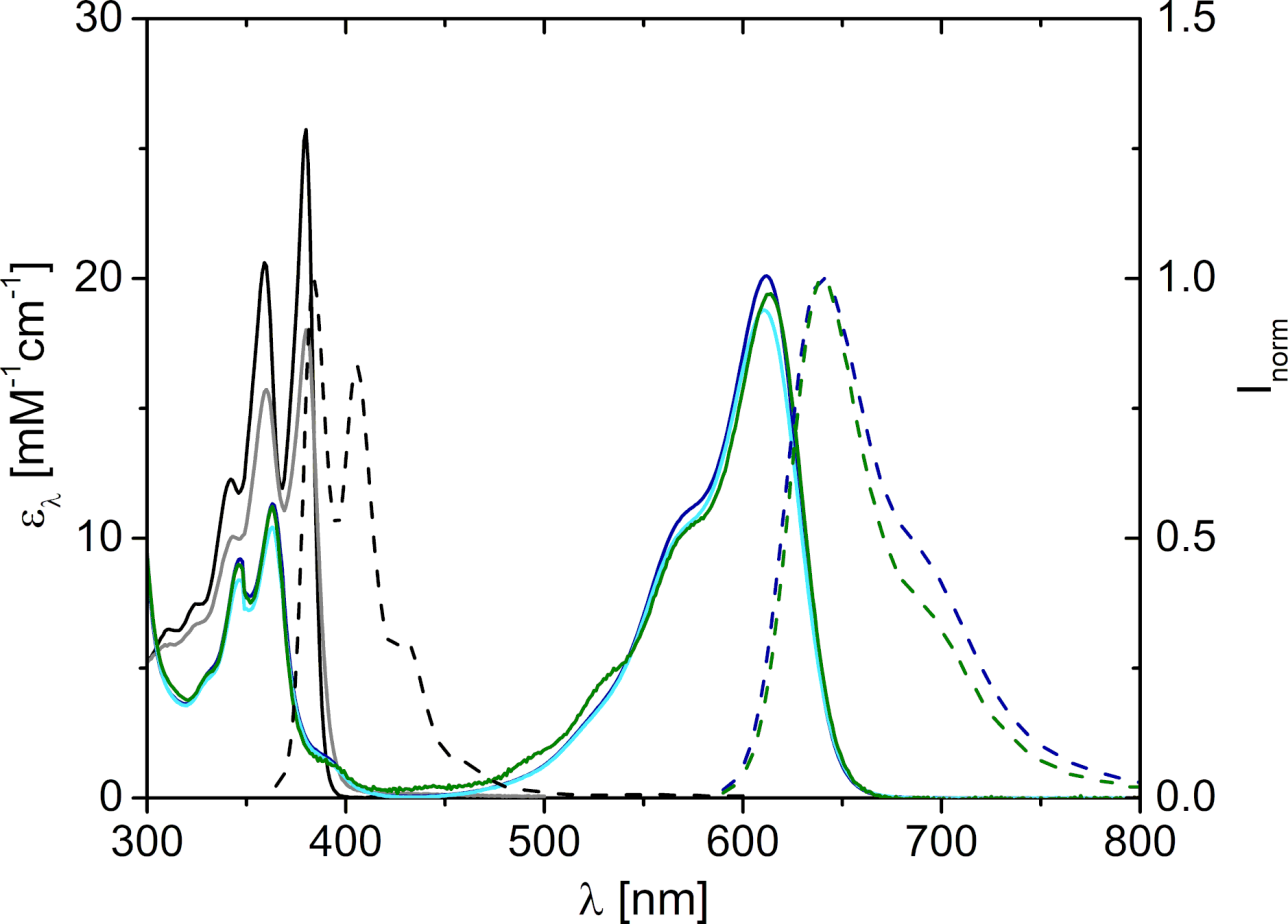
Optical properties of NDI **1** and cNDIs **2** and **6**: UV–vis absorbance in CH_2_Cl_2_ and in DMF (normal lines), normalized fluorescence in CH_2_Cl_2_ (dashed lines); **1** in CH_2_Cl_2_ (black), **1** in DMF (gray), **2** in CH_2_Cl_2_ (green), **6** in CH_2_Cl_2_ (dark blue), **6** in DMF (light blue), *l*_exc_ = 350 nm for **1**, λ_exc_ = 600 nm for **2** and **6**.

The synthesized reference NDI **1** and the new cNDI **6** as potential photoredox catalyst were characterized in DMF and CH_2_Cl_2_ by means of optical spectroscopy and electrochemistry. The UV–vis absorbance of the core-unsubstituted NDI **1** in CH_2_Cl_2_ show characteristic bands in the range between 300 nm and 400 nm with the maximum at 380 nm (Figure 1). The absorbance of NDI **1** in DMF is very similar to that in CH_2_Cl_2_, only the extinction is slightly reduced. The charge transfer character of the excited state of cNDI **6** should yield an absorbance in the visible range for photoredox catalysis. In fact, the absorbance of the new cNDI **6** in CH_2_Cl_2_ shows considerably red-shifted bands in the range between 500 nm and 650 nm with the maximum at 612 nm. The absorbance of **6** in DMF is very similar to that in CH_2_Cl_2_. The similarities of the UV–vis absorbance of the cNDIs **2** and **6** in CH_2_Cl_2_ exemplarily evidences that length and branching of the alkyl substituents both at the imide nitrogens and at the core amino groups have only little influence on the optical properties but significantly modulate the solubility in DMF. This result agrees with other cNDIs in literature [47–48,58]. NDI **1** shows weak fluorescence in CH_2_Cl_2_ with a maximum at 384 nm and a quantum yield of 7%. The Stokes’ shift is small (413 cm^−1^). Based on these values, the excitation energy *E*_00_ for the singlet state which is an important prerequisite for photoredox catalysis with **1** can be estimated to be 3.25 eV. In DMF, the fluorescence of **1** is completely quenched. This is due to a photoinduced charge transfer between NDI **1** and DMF. DMF has an oxidation potential of 0.38 V vs SCE [59]. Together with the reduction potential of *E*_red_ = 0.69 V and *E*_00_ = 3.25 eV for NDI **1** (vide infra), this electron transfer is clearly exergonic (Δ*G* = *E*_ox_ − *E*_red_ − *E*_00_ = −2.2 eV). NDI **6** shows a strong and broad fluorescence in CH_2_Cl_2_ with a maximum at 640 nm and a quantum yield of 48%. The Stokes’ shift is rather large (715 cm^−1^). The excitation energy *E*_00_ of cNDI **6** in the singlet state is approximately 1.98 eV and is significantly smaller than that of NDI **1** due to the visible light excitation which delivers less energy to the excited state.

The redox potentials of the reference NDI **1** and the cNDI **2** (due to the better solubility) were determined in comparison by cyclic voltammetry in CH_2_Cl_2_ and in the presence of 0.1 M *n*-Bu_4_PF_6_ as conducting salt (see Supporting Information File 1). The cyclic voltagram of NDI **1** shows two reversible reductions and one irreversible oxidation with *E* ≈ 0.85 V vs SCE. The two potentials in the negative potential range can be assigned to the formation of the radical anion **1^•−^**, *E*_½_(**1**/**1^•−^**) = −0.69 V, and the dianion **1^2−^**, *E*_½_(**1^•−^**/**1^2−^**) = 1.10 V (Table 1). These values agree well with literature results [48–49,54]. In comparison, the reductions of cNDI **2** to the radical anion **2^•−^** were observed at *E*_½_(**2**/**2^•−^**) = −1.06 V and to the dianion **2^2−^** at *E*_½_(**2^•−^**/**2^2−^**) = −1.50 V and show the electronic effect of the two *n*-propylamino substituents. Additionally, two reversible oxidations were observed at *E*_½_(**2^•+^**/**2**) = +0.99 V and *E*_½_(**2^2+^**/**2^•+^**) = +1.40 V which can be assigned to the electron-donating effect of the *n*-propylamino groups. The key values for the photoredox catalytic activity (vide infra) are the excited state potential for the reduction, E*_red_(2*/**2^•−^**) = +0.92 V, and for the oxidation E*_ox_(**2^•+^**/2*) = −0.99 V. According to the categories for strength of chemical redox agents [60], cNDI **2** in the excited state is a strong oxidant and a mild reductant. We assume based on literature-known cNDIs that the different alkyl groups of the other cNDIs **3**–**6** have no or only very little influence on the electrochemical properties in comparison with those of cNDI **2** [48–49]. Hence, the photoredox properties of the new cNDI **2**–**6** are comparable to those of eosin Y and rhodamine 6G as other organic photoredox catalysts.

**Table 1:** Optical and electrochemical properties of NDI **1** and cNDI **2** (in CH_2_Cl_2_) in comparison to other organic photoredox catalyst **X**, in particular eosin Y (EY), rhodamine 6G (Rh6G) and 9-mesityl-10-methylacridinium perchlorate (MesAcr) 1,2,3,5-tetrakis(carbazol-9-yl)-4,6-dicyanobenzenes (4CzIPN) and *N*-phenylphenothiazine (Ptz).

**X**	λ	*E*_00_^a^	*E*_½_(**X**/**X^•−^**)	E*_red_(X*/**X^•−^**)^a^	*E*_½_(**X^•+^**/**X**)	E*_ox_(**X^•+^**/X*)^a^
	[nm]	[eV]	[V]	[V]	[V]	[V]

**1**	380	3.25	−0.69	+2.56	–	–
**2**	612	1.98	−1.06	+0.92	+0.99	−0.99
EY [61]	539^b^	2.31	−1.08^c^	+1.23	+0.76^c^	−1.58
Rh6G [62]	530^d^	2.32	−1.14	+1.18	+1.23	−1.09
MesAcr [63]	425	2.67^e^	−0.49	+2.08^e^	–	–
Ptz [29]	320	3.25	–	–	+0.75	−2.50
4CzIPN [28]	≈370^b^	2.67	−1.24^b^	+1.43	+1.49^b^	−1.18

All potentials were converted from the ferrocene scale to the SCE scale [64]. ^a^For singlet state. ^b^In MeCN. ^c^In MeOH. ^d^In EtOH. ^e^CT state.

### Photoredox catalysis with NDI **1** and cNDI **6**

**Scheme 2:**
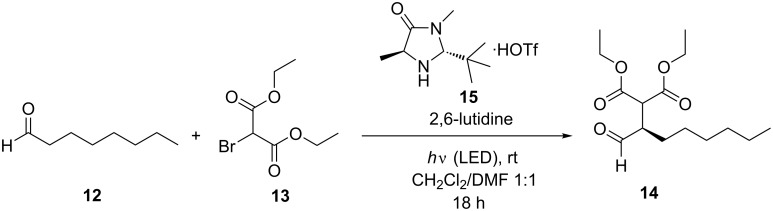
Photocatalytic α-alkylation of octanal (**12**): 500 mM **12**, 250 mM **13**, 50 mM (20 mol %) organocatalyst **15**, 500 mM 2,6-lutidine, NDI **1** or cNDI **2**–**6** as photoredox catalyst in 1.3 mL solvent, stirring, irradiation by LED, see Table 2.

The α-alkylation of 1-octanal (**12**) by diethyl 2-bromomalonate (**13**) yielding product **14** (Scheme 2) is one of the benchmark reactions for photoredox catalysis because it combines photoredox catalysis with organocatalysis [53]. Initially, [Ru(bpy)_3_]Cl_2_ was applied by MacMillan et al. as photoredox catalyst together with the chiral imidazolidinone **15** as organocatalyst to achieve enantioselectivity. Several proposal for the mechanism are found in literature ranging from a closed photoredox catalytic cycle [53,61] to a chain propagation mechanism with photoredox initiation [65]. We evaluated the reference NDI **1** and the cNDI **6** as new photoredox catalysts using this benchmark reaction. The samples were degassed by the freeze-pump-thaw method, the catalysis was performed under inert gas conditions (Argon), and the chemical conversions and yields were determined by ^1^H NMR spectroscopy (see Supporting Information File 1). The enantiomeric excess of the product **14** was determined after conversion with (2S,4S)-(+)-pentanediol into diastereomers and by integration of the corresponding signals in the ^1^H NMR spectrum. In all experiments with NDI **1** the enantiomeric excess exceeded a value of 78%; in all experiments with cNDI **6** the enantiomeric excess was higher than 81%. This parameter is omitted for clarity in the following paragraphs because enantioselectivity is not a matter of discussion in this work.

The photoredox catalysis with NDI **1** was performed by LEDs with 387 nm maximum emission wavelength and an irradiation time of 18 h. In order to ensure solubility of all components, a solvent mixture of DMF/CH_2_Cl_2_ = 1:1 was used. 20 mol % of organocatalyst **15** were applied. 2,6-Lutidine was added as base to trap protons that are potentially formed during the reaction and to ensure enamine formation with the organocatalyst **15**. Under these conditions, a moderate conversion of **13** (65%) and a low yield of product **14** (25%) were obtained (Table 2). In pure CH_2_Cl_2_ as solvent there was only a small conversion, but no product **14** detectable. Only the debrominated diethyl malonate was identified as side product. This makes conclusively clear that the solvent DMF is needed for this type of photoredox reaction. The control reaction without light did not show conversion at all. Control reactions with light, but without NDI **1** showed, however, a low conversion of 24% and a low yield of 18%. This is due to the UV-A absorption of the enamine that is formed as intermediate between 1-octanal (**12**) and the organocatalyst **15**. Similar photochemical reactions by direct excitation of the emanine intermediate were described by Melchiorre et al. [66]. In pure DMF this effect is even stronger and increases the conversion to 80% and the yield to 58% even in the absence of NDI **1**. Obviously, photoredox catalytically driven conversion by NDI **1** competes with the direct excitation of the intermediate enamine. This is the reason why lowering the concentration of the photoredox catalyst **1** from 5 mol % over 2.5 mol % to 1.25 mol % did not reduce conversions and yields, but increasing the amount of organocatalyst **15** from 20 mol % to 40 mol % finally improved the conversion to 99% and the yield to 60% Table 2, entry 5).

**Table 2:** Photoredox catalytic conversions of diethyl 2-bromomalonate (**13**) with 1-octanal (**12**) and yields of product **14** by different photoredox catalysts (**X**).

Entry	**X**	mol %	LED	solvent	Conversion (%) of **13**	Yield (%) of **14**

1	**1**	5	387	DMF/CH_2_Cl_2_ 1:1	65	25
2^a^	**1**	5	387	DMF/CH_2_Cl_2_ 1:1	87	45
3	**1**	2.5	387	DMF/CH_2_Cl_2_ 1:1	67	32
4	**1**	1.25	387	DMF/CH_2_Cl_2_ 1:1	67	30
5^a^	**1**	1.25	387	DMF/CH_2_Cl_2_ 1:1	99	60
6	–	–	387	DMF/CH_2_Cl_2_ 1:1	24	18
7	**1**	5	387	CH_2_Cl_2_	30	n.d.
8	**1**	5	387	DMF	69	44
9	–	–	387	DMF	80	58
10	**6**	0.1	520	DMF/CH_2_Cl_2_ 1:1	77	69
11	**6**	0.1	597	DMF/CH_2_Cl_2_ 1:1	46	43
12	**6**	0.1	637	DMF/CH_2_Cl_2_ 1:1	n.d.	n.d.

^a^40 mol % **15**.

**Figure 2:**
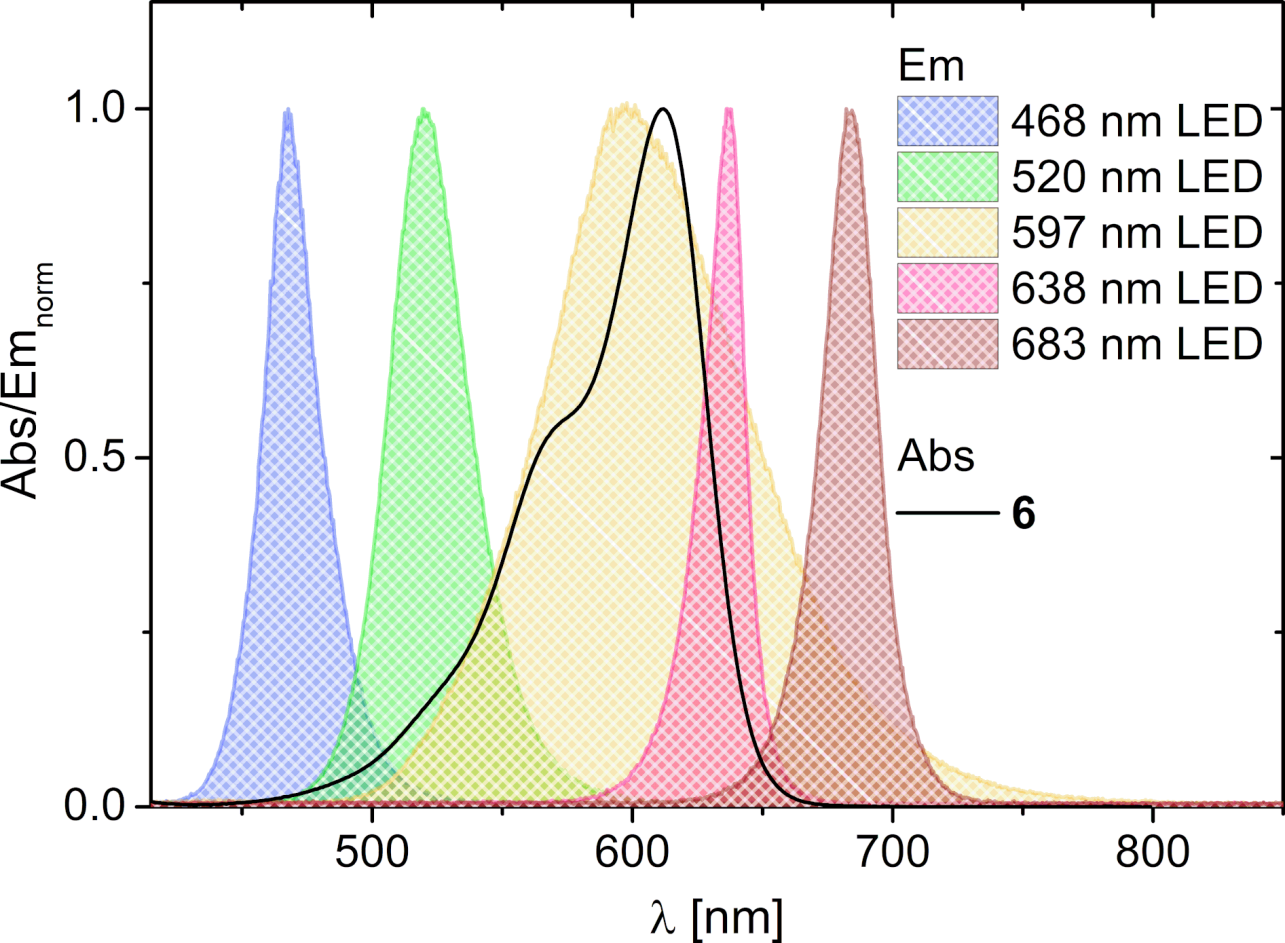
Normalized absorbance of cNDI **6** in comparison to normalized emission of the 468 nm, 520 nm, 597 nm, 638 nm and 683 nm LEDs.

These photoredox catalytic experiments with NDI **1** made obvious that the excitation must be shifted from the UV-A range into the visible range in order to achieve selective excitation of the photoredox catalyst and not a mixture of different photomechanisms. cNDI **6** with its absorbance between 500 nm and 650 nm and a maximum at 612 nm fullfills this requirement. The corresponding photoredox catalytic experiment with cNDI **6** and the 597 nm LED as irradiation source gives a conversion of 46% and a product yield of 43%. The emission of this LED overlays well with the absorbance of cNDI **6** (Figure 2). As expected, the photoredox catalytic reaction with cNDI **6** is much “cleaner” than with NDI **1** and the substrate conversion differs only slightly from the product yield. Taken together, cNDI **6** is a suitable visible light photoredox catalyst for this reaction although its excited state potential (comparable to E*_red_(2*/**2^•−^**) = +1.92 V) is much lower than that of NDI **1** (E*_red_(1*/**1^•−^**) = +2.56 V) but obviously still sufficiently high. However, irradiations at different wavelengths gave surprising results: (i) If the 637 nm LED is applied for irradiation, there is no conversion of substrate **13** detectable although the emission of this LED also overlays well with the absorbance of cNDI **6**. (ii) If the 520 nm LED was applied the conversion increased to 77% and the yield to 69%, although excitation of only the side absorption band of cNDI **6** is realized in this experiment. Obviously, there is a strong difference between the absorbance of cNDI **6** and its photoredox catalytic activity profile with respect to the irradiation wavelength which was further studied by more detailed kinetic measurements.

**Figure 3:**
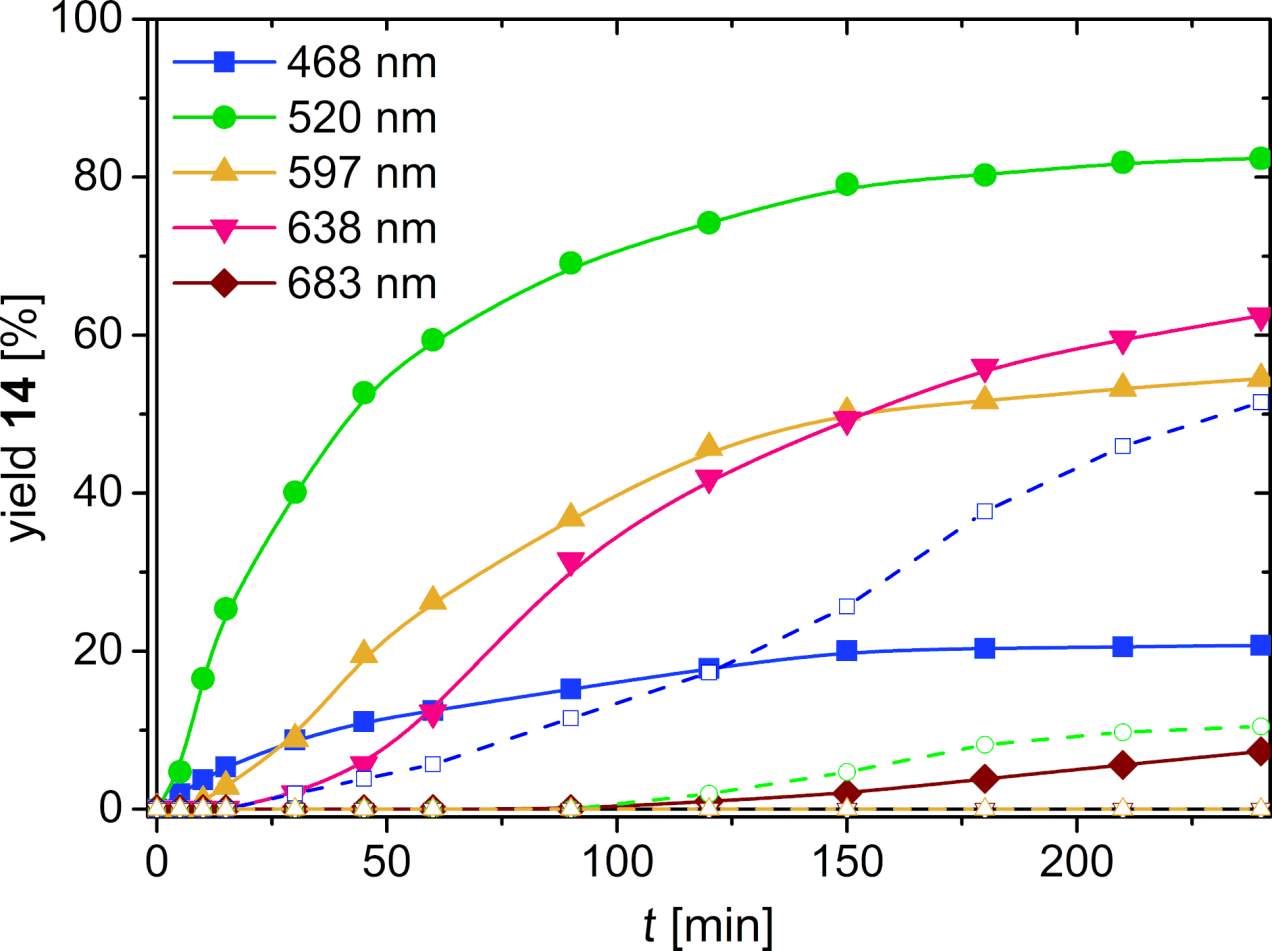
Kinetic analysis of yields of product **14** in the presence (solid lines) and in the absence (dashed lines) of cNDI **6**: 500 mM **12**, 250 mM **13**, 50 mM organocatalyst **15**, 500 mM 2,6-lutidine in 1.3 mL DMF, stirring, irradiation by LEDs.

Time-dependent product analysis was performed during the photoredox catalytic conversions using different LEDs for irradiations at 468 nm, 520 nm, 597 nm, 638 nm and 683 nm (see LED emissions in Figure 2, results in Figure 3). These experiments were performed in pure DMF, which further accelerates the photoredox catalytic conversion due to the better solubility of the cNDI **6**. The product formation is almost finished after 4 h of irradiation. The most productive excitation provides the 520 nm LED with a product yield of 82% although the emission of this LED overlays only partially with the absorbance of cNDI **6**. The emissions of the 597 nm and 638 nm LEDs do overlay better but show lower yields of 55% and 62%, respectively, in photoredox catalysis. The lag phases of more than 30 min in the latter experiments indicate that indeed the enamine formation plays a crucial role. According to the mechanism suggested by Yoon et al. the photoredox catalytic cycle is initiated by the oxidation of the enamine [65]. Control experiments were performed without cNDI **6** as photoredox catalyst to elucidate product formation by direct excitation of the intermediate enamine, as discussed above. In fact, by irradiation at 468 nm there is a significant amount of product formed without cNDI **6** (52% yield) that even exceeds the product formation in the presence of cNDI **6** (21% yield). By irradiation at 520 nm there is only a small amount (10% yield) of product **14** formed without cNDI **6**, but only after longer irradiation times (approximately after 2 h). Irradiations at 597 nm and 638 nm are completely unproductive without the cNDI **6** as photoredox catalyst. It is obvious that the absorbance of cNDI **6** differs from the photoredox catalytic activity profile. This activity profile shows highest values at 520 nm whereas the absorbance has the maximum at 612 nm. The observed spectral sensitivity of the photoredox catalytic product formation cannot be easily explained. One possible reason could be the different luminous flux of the applied high-power LEDs. Given that Kasha’s rule is also applicable for photochemical reactions and not only fluorescence this result is probably a “non-Kasha” photophysical dynamic behavior which can be found also for other photochemical reactions in the literature [67], but needs further investigations by time-resolved spectroscopy. The irradiations at 638 nm and 597 nm overlap with the emission range of cNDI **6** so that an inner filter effect cannot be excluded. Taken together, it makes clear that tuning of optical and electrochemical properties of potential photoredox catalysts have to be combined with their elucidation of their photophysical dynamic behavior.

## Conclusion

The cNDIs **2**–**6** were synthesized as potential photoredox catalysts that differ by the alkyl groups at the imide nitrogen and at the two amino substituents at the core in order to improve their solubility. Especially the cNDI **6** showed a good solubility in DMF that is comparable to the unsubstituted NDI **1** and suitable for photoredox catalysis in solvent mixtures with DMF. Due to the charge-transfer character in the excited state the absorbances of cNDIs **2** and **6** are shifted into the visible range with a broad band between 500 nm and 650 nm. The reduction potential to form the radical anion of such a cNDI is significantly shifted to a more negative potential of *E*_½_(**2**/**2^•−^**) = 1.06 V. Together with *E*_00_ = 1.98 eV an excited state potential of E*_red_(2*/**2^•−^**) = +0.92 V was estimated for the singlet state which renders such cNDIs to be suitable to photocatalyze organic reactions. The photoredox catalytic activities of NDI **1** and cNDI **6** in comparison were successfully evaluated for the MacMillan benchmark reaction. This photoredox catalytic reaction in the presence of cNDI **6** was much “cleaner” than with NDI **1** since the conversions differed only slightly from the product yields. Irradiations were performed with LEDs in the visible light range between 520 nm and 640 nm. The substrate conversion and product yields were significantly higher by LED irradiation into the absorbance shoulder at 520 nm which implies a non-Kasha-type photodynamic behavior. This makes clear that future photoredox catalysts must not only by tuned by their optical and electrochemical properties but also by their photophysical dynamics. To the best of our knowledge this is the first report on the usage of cNDIs as photoredox catalysts. The irradiation by visible light from LEDs as energy-saving light sources together with the use of an organic dye instead of a transition metal complex as photoredox catalyst improve the sustainability and make photoredox catalysis “greener”.

## Experimental

All experimental details are described in the Supporting Information File 1.

## Supporting Information

File 1 Synthetic protocols, copies of ^1^H and ^13^C NMR spectra, mass spectra, and cyclic voltammetry data.
