# Supplementary material for: Naphthalene diimides with improved solubility for visible light photoredox catalysis
Source: Beilstein J Org Chem. 2019 Aug 27;15:2043–51. doi: 10.3762/bjoc.15.201 (PMC6720061; doi:10.3762/bjoc.15.201)
Supplement: File 1 — Synthetic protocols, copies of 1H and 13C NMR spectra, mass spectra, and cyclic voltammetry data. [file Beilstein_J_Org_Chem-15-2043-s001.pdf]

BEILSTEIN JOURNAL OF ORGANIC CHEMISTRY

Supporting Information

for

Naphthalene diimides with improved solubility for visible light photoredox catalysis

Barbara Reiß and Hans-Achim Wagenknecht

Beilstein J. Org. Chem. **2019,** *15,* 2043–2051. doi:10.3762/bjoc.15.201

Synthetic protocols, copies of ^{1}H and ^{13}C NMR spectra, mass spectra, and cyclic voltammetry data

License and Terms: This is a supporting information file under the terms of the Creative Commons Attribution License (http://creativecommons.org/licenses/by/4.0). Please note that the reuse, redistribution and reproduction in particular requires that the authors and source are credited.
The license is subject to the *Beilstein Journal of Organic Chemistry* terms and conditions: (https://www.beilstein-journals.org/bjoc)

Content

Materials and methods S2

Synthetic procedures S3

Images of ^{1}H NMR spectra, ^{13}C NMR spectra and mass spectra S9

Photoredox catalytic experiments S21

Cyclic voltammetry S24

References S25

Materials and methods

Chemicals and dry solvents were purchased from commercial sources and were used without further purification unless otherwise stated. Solvents of purity grade p.a. were used for syntheses and photocatalyses. All inert gas syntheses and photocalyses were performed using argon (99.999%). The inert photoredox catalytic experiments were degassed by the freeze-pump-thaw technique. NMR spectroscopic data was recorded using the spectrometer Bruker Ascend 500 ^{1}H NMR (500 MHz), ^{13}C NMR (126 MHz). Chemical shifts of the ^{1}H and ^{13}C NMR spectra are reported in parts per million (ppm) relative to the solvent as an internal standard and was converted to the TMS-reference. Routine ^{13}C NMR spectroscopy was recorded while applying broadband ^{1}H- decoupling. Coupling constants (*J*) are given in Hertz (Hz) and the multiplicity of signals are reported as followed: s (singlet), d (doublet), t (triplet), sext (sextet), m (multiplet), br (broad singlet), dt (doublet of triplets), td (triplet of doublets). Optical measurements were carried out using a Varian Cary 100 spectrometer at room temperature and a HORBIA-Scientific Fluoromax 4 spectrofluorometer with an AC 200 thermostat from Thermo Scientific and the FluoroEssence software V3.5 in semi-micro quartz glass cuvettes (width 1 cm, volume 1.4 mL) from Starna. The fluorescence quantum yields were determined by the Quantaurus QY C11347 from Hamatsu. The cyclic voltammetry measurements were carried out with complete exclusion of air and moisture. The working electrode and the counter electrode were made of platinum. Reference electrode serving as the potential zero point was made of silver. For the electrolysis solution, a 0.05 M solution of tetrabutylammonium hexafluorophosphate in dry CH_2Cl_2 was prepared.

Synthetic procedures

Syntheses of NDI 1, cNDI 8 and organocatalyst 15. NDI **1** was synthesized according to the literature [S1]. The intermediate cNDI **8** was synthesized according to the literature and used as crude material [S2]. The organocatalyst **15** was synthesized according to the literature [S3] and used after treatment with Ag(OTf) as triflate salt.

Synthesis of cNDI 9. cNDI **8** (1.47 mg, 3.45 mmol, 1.00 equiv) were suspended in CH_2Cl_2 (300 mL) and *n*-octylamine (1.21 mL, 946 mg; 7.32 mmol, 2.12 equiv) was added. The mixture was refluxed for 2 h. PBr_3 (0.97 mL, 2.80 g, 10.4 mmol, 3.00 equiv) was slowly and dropwise added. The mixture was refluxed for 1 h. After cooling down to rt, the mixture was poured into water (300 mL) and stored overnight. The aqueous phase was extracted two times with CH_2Cl_2. The combined organic phases were dried with Na_2SO_4. The solvent was removed under vacuum. The product was purified by column chromatography (SiO_2, hexane/CH_2Cl_2 1:3, ø 4 cm, 14 cm). 940 mg (1.45 mmol, 42%) of a light yellow solid material were isolated. The spectroscopic data of cNDI **9** was identical to the literature [S4].

Synthesis of cNDI 10. cNDI **8** (400 mg, 0.94 mmol, 1.00 equiv.) was suspended in CH_2Cl_2 (35 mL) and 5-methylhexane-2-amine (0.3 mL, 227 mg; 1.97 mmol, 2.12 equiv) was added. The mixture was refluxed for 2 h. PBr_3 (5.64 mL 1 M in CH_2Cl_2, 1.53 g, 5.64 mmol, 6.00 equiv) was slowly and dropwise added. The mixture was refluxed for 1 h. After cooling to rt, the mixture was poured into water (100 mL) and stored overnight. The aqueous phase was extracted two times with CH_2Cl_2. The combined organic phases were dried with Na_2SO_4. The solvent was removed under

vacuum. The product was purified by column chromatography (SiO_2, hexane/CH_2Cl_2 1:3, ø 4 cm, 14 cm). 81 mg (130 µmol, 14%) of a light yellow solid material were isolated. T.l.c. (hexane: CH_2Cl_2 1:3): R_f = 0.52. ^{1}H-NMR (500 MHz, $CDCl_3$): δ [ppm]=8.96 (s, 2H, Ar H), 5.20 (dt, *J* = 8.1, 6.6 Hz, 2H, -NCH-), 2.11–2.03 (m, 2H, -CHC**H**$_2$-), 1.91–1.83 (m, 2H, -CHC**H**$_2$-), 1.53–1.45 (m, 8H, -C**H**(CH_2)$_3$, -NCHC**H**$_3$), 1.21–1.13 (m, 2H, -CHC**H**$_2$-), 1.04–0.97 (m, 2H, -CHC**H**$_2$-), 0.79 (t, *J* = 6.7 Hz, 12H, -CH_3). ^{13}C-NMR (126 MHz, $CDCl_3$): δ [ppm]=161.4, 161.3, 139.2, 128.2, 127.8, 125.6, 124.4, 51.7, 36.3, 31.4, 28.1, 22.8, 22.7, 18.3. MS (FAB): m/z [%]=617.1 [47.0], 618.1 [39.1], 619.1 [100.0, M^+], 620.1 [65.2], 621.1 [91.3], 622.1 [43.5]. HRMS (EI): found: m/z 619.0801 [M^+], calcd: 619.0802.

Synthesis of cNDI 11. cNDI **8** (400 mg, 0.94 mmol, 1.00 equiv) was suspended in CH_2Cl_2 (35 mL) and pentane-3-amine (0.23 mL, 173 mg, 1.98 mmol, 2.11 equiv) was added. The mixture was refluxed for 2 h. PBr_3 (5.64 mL 1 M in CH_2Cl_2, 1.53 g, 5.64 mmol, 6.00 equiv) was slowly and dropwise added. The mixture was refluxed for 1 h. After cooling to rt, the mixture was poured into water (100 mL) and stored overnight. The aqueous phase was extracted two times with CH_2Cl_2. The combined organic phases were dried with Na_2SO_4. The solvent was removed under vacuum. The product was purified by column chromatography (SiO_2, hexane/CH_2Cl_2 1:3, ø 4 cm, 14 cm). 124 mg (0.22 mmol, 24%) of a light yellow solid material were isolated. Column chromatography. T.l.c. (hexane: CH_2Cl_2 1:1): R_f = 0.42. ^{1}H-NMR (500 MHz, $CDCl_3$): δ [ppm]=8.97 (s, 4H, Ar-H), 5.02 (tt, J = 9.3, 6,0 Hz, 2H, -NCH-), 2.25-2.13 (m, 4H, -NCHC**H**$_2$-), 2.01-1.87 (m, 4H, -NCHC**H**$_2$-), 0.89 (t, J = 7.4 Hz, 12H, -CH_3). Due to the very low solubility no ^{13}C NMR and no MS data was obtained. The crude material was used for the synthesis of cNDI **6**.

Synthesis of cNDI 2. cNDI **9** (200 mg, 0.31 mmol, 1.00 equiv) was dissolved in *n*-propylamine (5 mL. 60.9 mmol) and refluxed for 2 h at 55 °C in a pressure vial. The amine was removed under vacuum. The solid material was dissolved in CH_2Cl_2 (10 mL) and treated with 1 N aqueous HCl (10 mL). The aqueous phase was extracted two times with CH_2Cl_2 (each 10 mL). The combined organic phases were washed with water (20 mL) and dried with Na_2SO_4. The solvent was removed under vacuum. The product was purified by column chromatography (SiO_2, hexane/CH_2Cl_2 3:1→1:2, ø 4 cm, 12 cm). The final products were dissolved in benzene, lyophilized and dried under vacuum. 149 mg (0.24 mmol, 80%) of a blue-purple solid material were isolated. T.l.c. (hexane:$CH_2Cl_2$2:3): R_f = 0.28. ^{1}H NMR (500 MHz, $CDCl_3$): δ [ppm]=9.32 (t, *J* = 5.3 Hz, 2H, NH), 8.05 (s, 2H, Ar-H), 4.13 (t, J = 7.6 Hz, 4H, O=C-NCH_2-), 3.44 (td, *J* = 7.2, 5.1 Hz, 4H, Ar-NCH_2), 1.87–1.79 (m, 4H, O=C-$NCH_2C\mathbf{H}_2$), 1.70 (tt, *J* = 8.2, 7.7 Hz, 4H, Ar-$NCH_2C\mathbf{H}_2$), 1.47-1.22 (m, 20H, CH_2), 1.11 (t, *J* = 7.4 Hz, 6H, $NCH_2CH_2C\mathbf{H}_3$), 0.86 (t, *J* = 6.6 Hz, 6H, CH_3). ^{13}C NMR (126 MHz, $CDCl_3$): δ (ppm)= 166.3, 163.1, 149.2, 125.8, 121.1, 118.3, 101.8, 45.0, 40.6, 32.0, 29.5, 29.4, 28.3, 27.4, 22.9, 22.8, 14.3, 11.9.
MS (EI): m/z [%]=604.3 [100.0, M^+], 605.3 [43.3], 606.3 [10.1]. HRMS (EI): found: m/z 604.3981 [M^+], calcd: 604.3983.

Synthesis of cNDI 3. cNDI **10** (30 mg, 0.05 mmol, 1.00 equiv) was dissolved in *n*-propylamine (2 mL, 24.4 mmol) and refluxed for 2 h at 55 °C in a pressure vial. The amine was removed under vacuum. The solid material was dissolved in CH_2Cl_2 (4 mL) and treated with 1 N aqueous HCl (10 mL). The aqueous phase was extracted two times with CH_2Cl_2 (each 4 mL). The combined organic phases were washed with water (10 mL) and dried with Na_2SO_4. The solvent was removed under vacuum. The

product was purified by column chromatography (SiO_2, hexane/CH_2Cl_2 2:1→3:2, ø 4 cm, 12 cm). The final products were dissolved in benzene, lyophilized and dried under vacuum. 24 mg (0.04 mmol, 87%) of a blue-purple solid material were isolated. T.l.c. (hexane:CH_2Cl_2 3:2): R_f = 0.46. ^{1}H-NMR (500 MHz, $CDCl_3$): δ [ppm]=9.34 (t, *J* = 5.4 Hz, 2H, -NH), 8.10 (s, 2H, Ar-H), 5.27-5.19 (m, 2H, -NCH-), 3.44 (dt, *J* = 7.1, 5.3 Hz, 4H, -$NHCH_2$-), 2.17-2.09 (m, 2H, -CHC**H**$_2$-), 1.93–1.86 (m, 2H, -CHC**H**$_2$-), 1.80 (sext, *J* = 7.3 Hz, 4H, -NCH_2C**H**$_2$-), 1.55–1.47 (m, 10H, -NCHC**H**$_3$, -CHC**H**$_2$-), 1.26–1.02 (m, 6H, -N(CH_2)$_2$C**H**$_3$), 0.82 (t, *J* = 6.9 Hz, 12H, -CH_3). ^{13}C-NMR (126 MHz, $CDCl_3$): δ [ppm]=167.1, 149.4, 121.4, 45.1, 36.4, 31.6 ,28.1, 22.9, 22.8, 22.7, 18.4, 11.8. MS (FAB): m/z [%]=573.4 [5.8], 574.4 [12.6], 575.4 [32.3], 576.4 [100.0, M^+], 577.4[74.1], 578.4 [25.1], 579.4 [5.9]. HRMS (EI): found: m/z 576.3669 [M^+], calcd: 576.3670.

Synthesis of cNDI 4. cNDI **8** (250 mg, 0.59 mmol, 1.00 equiv) was dissolved in 1-propylbutylamine (3 mL, 19.5 mmol) and refluxed for 2 h at 90 °C in a pressure vial. The amine was removed under vacuum. The solid material was dissolved in CH_2Cl_2 (10 mL) and treated with 1 N aqueous HCl (10 mL). The aqueous phase was extracted two times with CH_2Cl_2 (each 10 mL). The combined organic phases were washed with water (20 mL) and dried with Na_2SO_4. The solvent was removed under vacuum. The product was purified by column chromatography (SiO_2, hexane/CH_2Cl_2 2:1 +10% toluene, ø 3 cm, 16 cm). The final products were dissolved in benzene, lyophilized and dried under vacuum. 57 mg (0.08 mmol, 14%) of a blue-purple solid material were isolated. T.l.c. (hexane:CH_2Cl_2 2:1 + 10% toluene): R_f = 0.54. ^{1}H-NMR (300 MHz, $CDCl_3$): δ [ppm]=9.35 (br, 2H, -NH-), 8.14 (s, 2H, Ar-H), 5.22 (tt, *J* = 9.2, 5.9 Hz, 2H, -(O=C)$_2$NCH-), 3.91–3.84 (m, 2H, -NHC**H**-), 2.27–2.14 (m, 4H, -(O=C)$_2$NCHC**H**$_2$-), 1.84–1.78 (m, 4H, -(O=C)$_2$NCHC**H**$_2$-), 1.68–1.61 (m,

8H, -NHCHC**H**$_2$-), 1.45–1.25 (m, 16H, -C**H**$_2$CH$_3$-), 0.97–0.88 (m, 24H, -CH$_3$). ^{13}C-NMR (126 MHz, CDCl$_3$): δ [ppm] = 167.5, 167.1, 164.6, 163.5, 149.4.2, 126.5, 125.8, 121.4, 119.2, 118.4, 102.2, 101.5, 54.4, 53.4, 52.7, 37.6, 34.8, 34.5, 29.9, 22.9, 20.3, 19.3, 14.4, 14.2. MS (FAB): m/z (%)=685.5 [46.1], 686.5 [100], 687.5 [95.3], 688.9 [58.8], 689.5 [30.0, M$^+$].

Synthesis of cNDI 5. cNDI **8** (250 mg, 0.59 mmol, 1.00 equiv) was dissolved in 3-aminopentane (3 mL, 25.8 mmol) and refluxed for 2 h at 90 °C in a pressure vial. The amine was removed under vacuum. The solid material was dissolved in CH_2Cl_2 (10 mL) and treated with 1 N aqueous HCl (10 mL). The aqueous phase was extracted two times with CH_2Cl_2 (each 10 mL). The combined organic phases were washed with water (20 mL) and dried with Na_2SO_4. The solvent was removed under vacuum. The product was purified by column chromatography (SiO_2, hexane/CH_2Cl_2 1:1, ø 1 cm, 12 cm). The final products were dissolved in benzene, lyophilized and dried under vacuum. 85 mg (0.15 mmol, 25%) of a blue-purple solid material were isolated Column chromatography. T.l.c. (Hexan/DCM 2:1): R_f = 0.28. ^{1}H-NMR (500 MHz, CDCl$_3$): δ [ppm] = 9.37 (d, *J* = 8.2 Hz, 2H, -NH-), 8.15 (s, 2H, Ar-H), 5.07 (tt, *J* = 9.5, 6.1 Hz, 2H, -(O=C)$_2$NCH-), 3.78–3.71 (m, 2H, -NHC**H**-), 2.27–2.18 (m, 4H, -(O=C)$_2$NCHC**H**$_2$-), 1.95–1.90 (m, 4H, -(O=C)$_2$NCHC**H**$_2$-), 1.79–1.65 (m, 8H, -NHCHC**H**$_2$-), 1.00 (t, *J* = 7.4 Hz, 12H, -NHCHCH$_2$C**H**$_3$), 0.89 (t, *J* = 7.5 Hz, 12H, -(O=C)$_2$NCHCH$_2$C**H**$_3$). ^{13}C-NMR (126 MHz, CDCl$_3$): δ [ppm]=149.5, 131.1, 129.0, 121.5, 55.6, 45.1, 32.1, 29.9, 29.5, 27.5, 25.1, 22.9, 14.3, 11.9, 11.5, 10.4. MS (FAB): m/z [%] = 576.4 [100.0, M$^+$], 577.4 [85.5], 575.4 [29.6].
HRMS (EI): found: m/z 576.3671 [M$^+$], calcd: 576.3670.

Synthesis of cNDI 6. cNDI **11** (30 mg, 0.05 mmol, 1.00 equiv.) and *n*-propylamine (87 μL, 0.53 mmol, 10.0 equiv) were dissolved in DMF (1 mL) and refluxed for 2 h in a pressure vial. The amine was removed under vacuum. The solid material was dissolved in CH_2Cl_2 (2 mL) and treated with 1 N aqueous HCl (10 mL). The aqueous phase was extracted two times with CH_2Cl_2 (each 4 mL). The combined organic phases were washed with water (10 mL) and dried with Na_2SO_4. The solvent was removed under vacuum. The product was purified by column chromatography (SiO_2, hexane:CH_2Cl_2 1:1, ø 2 cm, 12 cm). The final products were dissolved in benzene, lyophilized and dried under vacuum. 16 mg (0.03 mmol, 58%) of a blue-purple solid material were isolated. T.l.c. (hexane:CH_2Cl_2 1:1): R_f = 0.52. ^{1}H-NMR (300 MHz, $CDCl_3$): δ [ppm]=9.37 (t, *J* = 7.6 Hz, 2H, -NH), 8.15 (s, 2H, Ar-H), 5.13–5.03 (m, 2H, -NCH-), 3.47 (dt, *J* = 7.3, 5.4 Hz, 4H, -NC**H**$_2$-), 2.31–2.16 (m, 4H, -NCH$_2$C**H**$_2$-), 1.98–1.78 (m, 8H, -NCH$_2$C**H**$_2$-), 1.09 (t, *J* = 7.4 Hz, 6H, -N(CH$_2$)$_2$C**H**$_3$), 0.89 (t, *J* = 7.4 Hz, 12H, -CHCH$_2$C**H**$_3$). ^{13}C-NMR (126 MHz, $CDCl_3$): δ [ppm]=149.9, 121.9, 100.5, 45.5, 30.3, 25.5, 23.4, 14.7, 12.3, 11.9. MS (FAB): m/z [%] = 520.3 [100.0, M^+], 521.3 [88.0], 522.3 [28.1], 523.3 [6.7]. HRMS (EI): found: m/z 520.3042 [M^+], calcd: 520.3044.

Images of ^{1}H NMR spectra, ^{13}C NMR spectra and mass spectra

cNDI 10

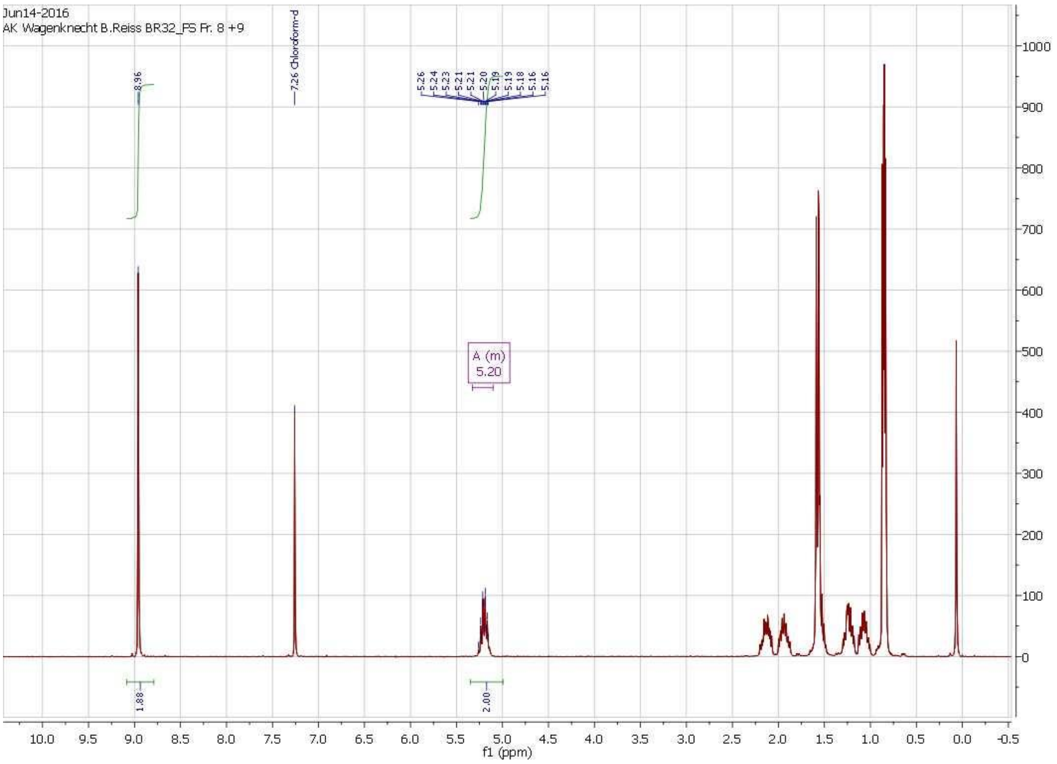

Figure S1. Image of ^{1}H NMR spectrum (500 MHz, $CDCl_3$).

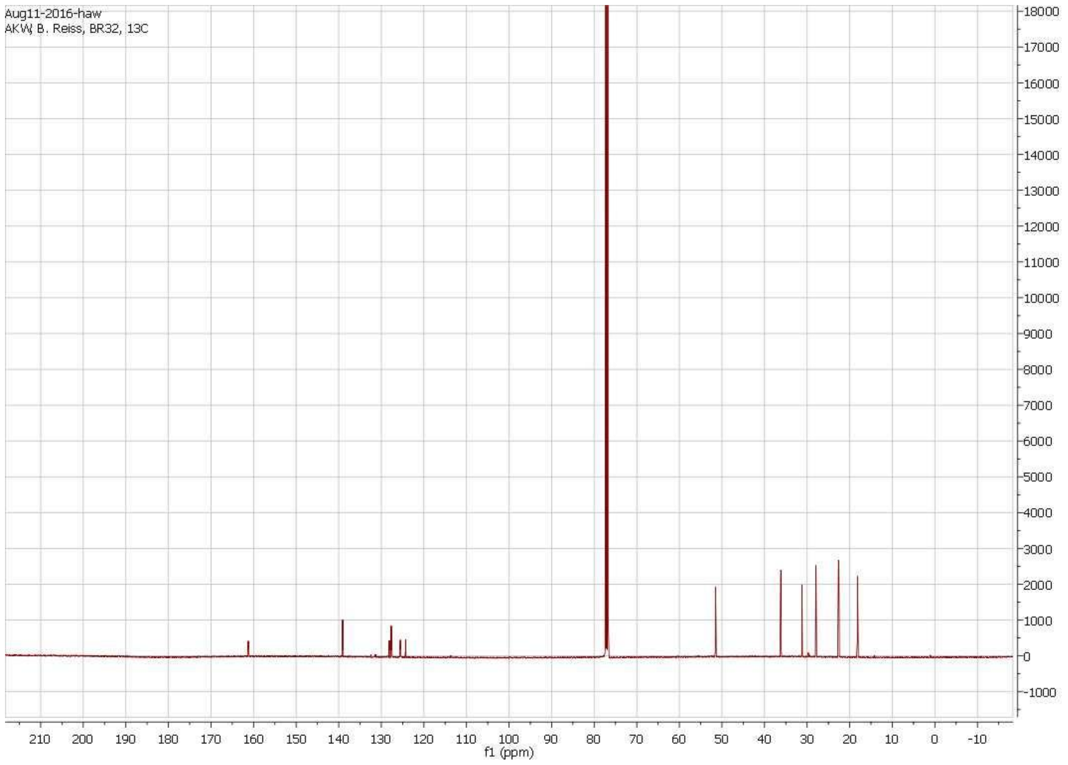

Figure S2. Image of ^{13}C NMR spectrum (126 MHz, $CDCl_3$).

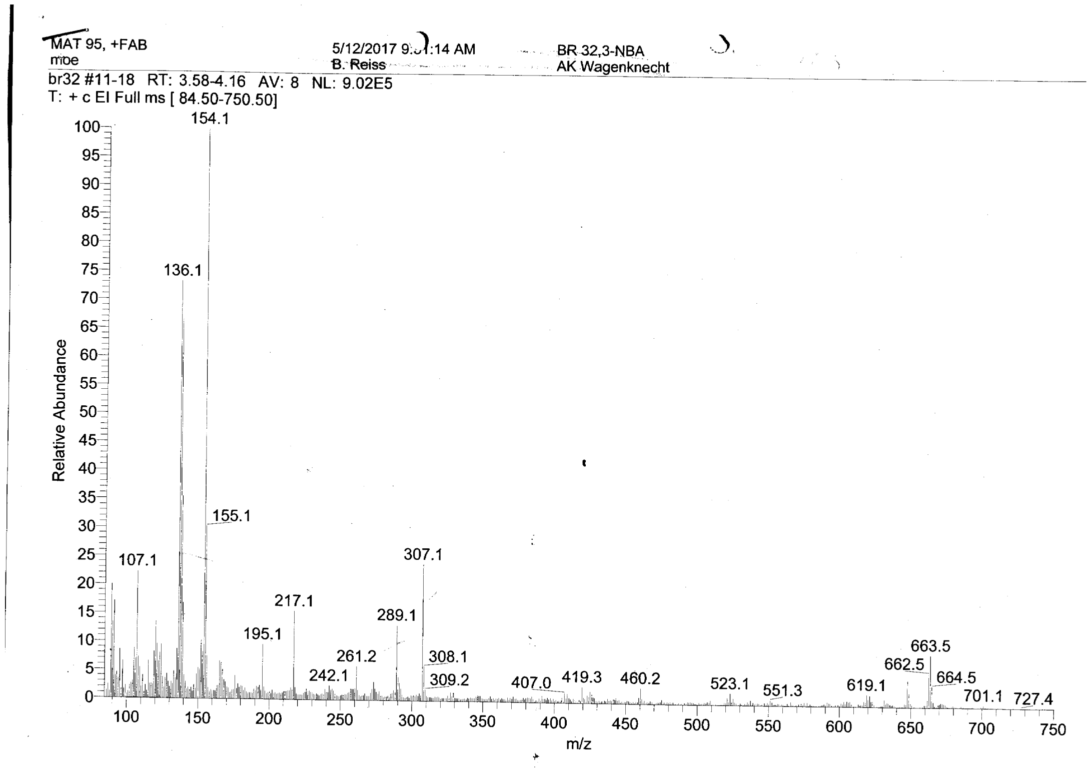

Figure S3. Image of mass spectrum (FAB).

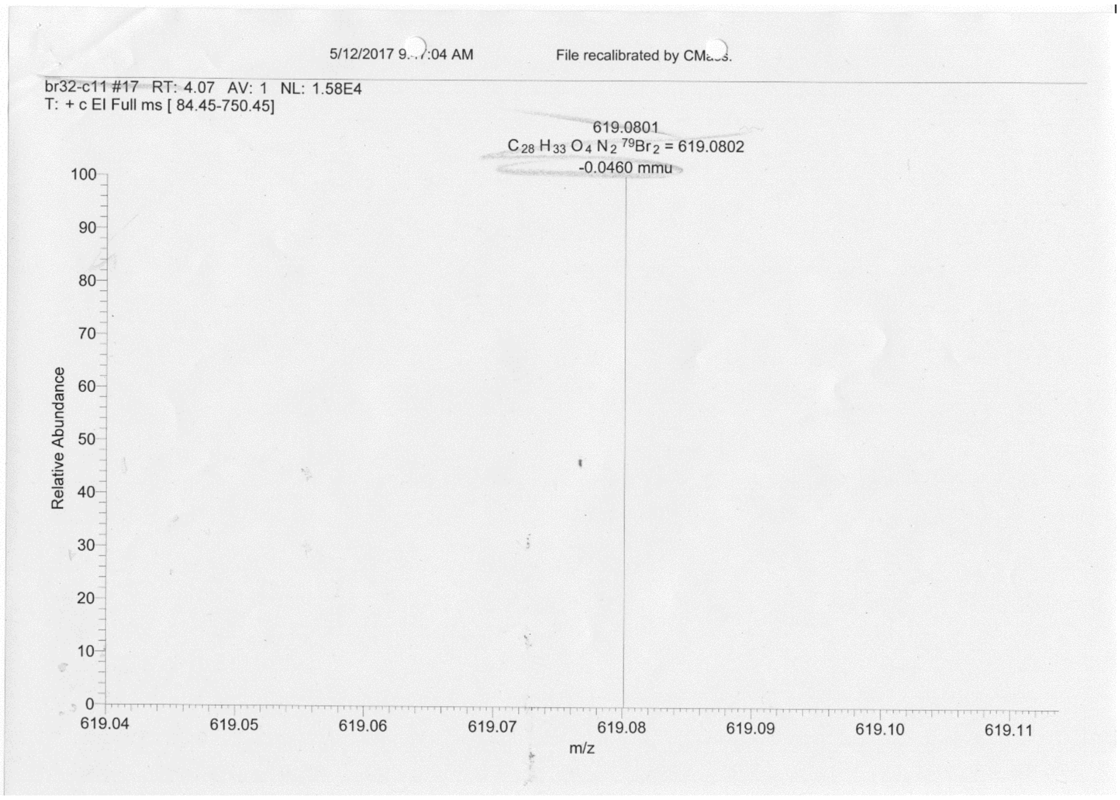

Figure S4. Image of HR mass spectrum (EI).

cNDI 11

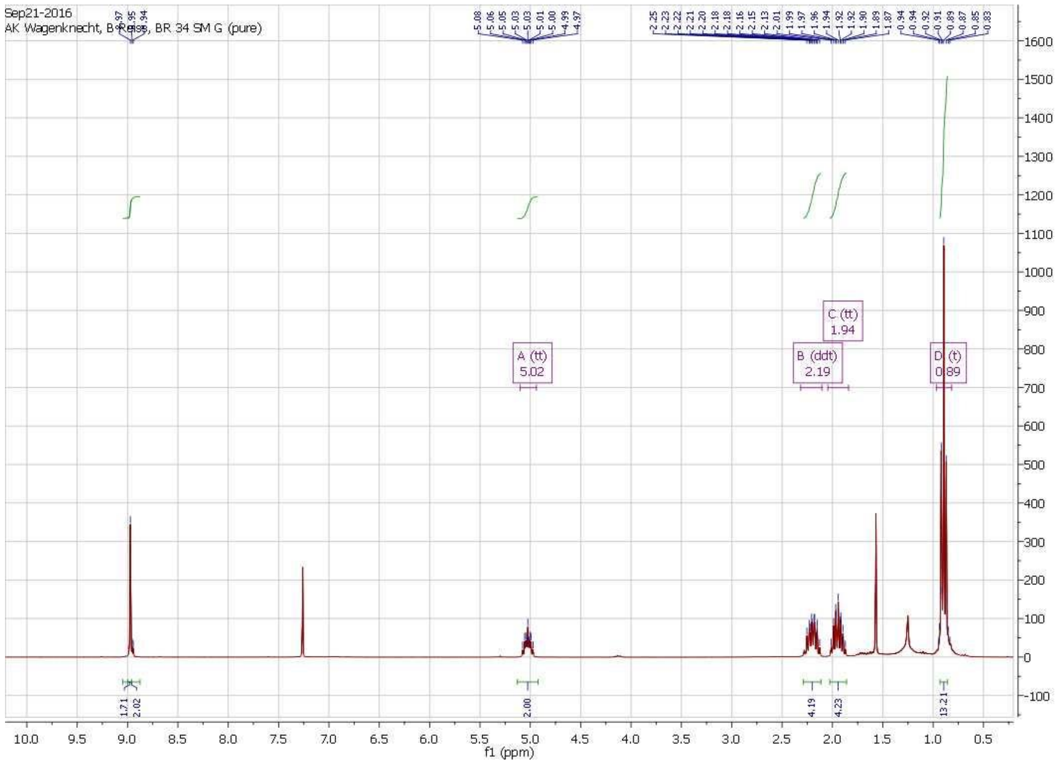

Figure S5. Image of ^1H NMR spectrum (500 MHz, $CDCl_3$).

cNDI 2

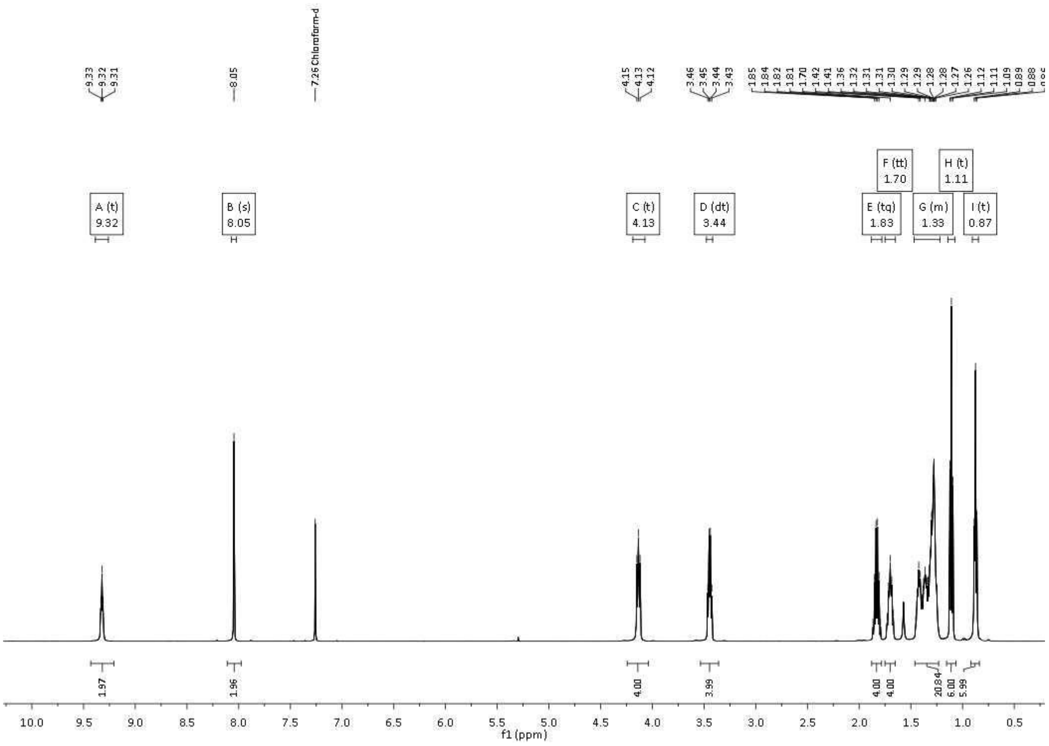

Figure S6. Image of ^{1}H NMR spectrum (500 MHz, $CDCl_3$).

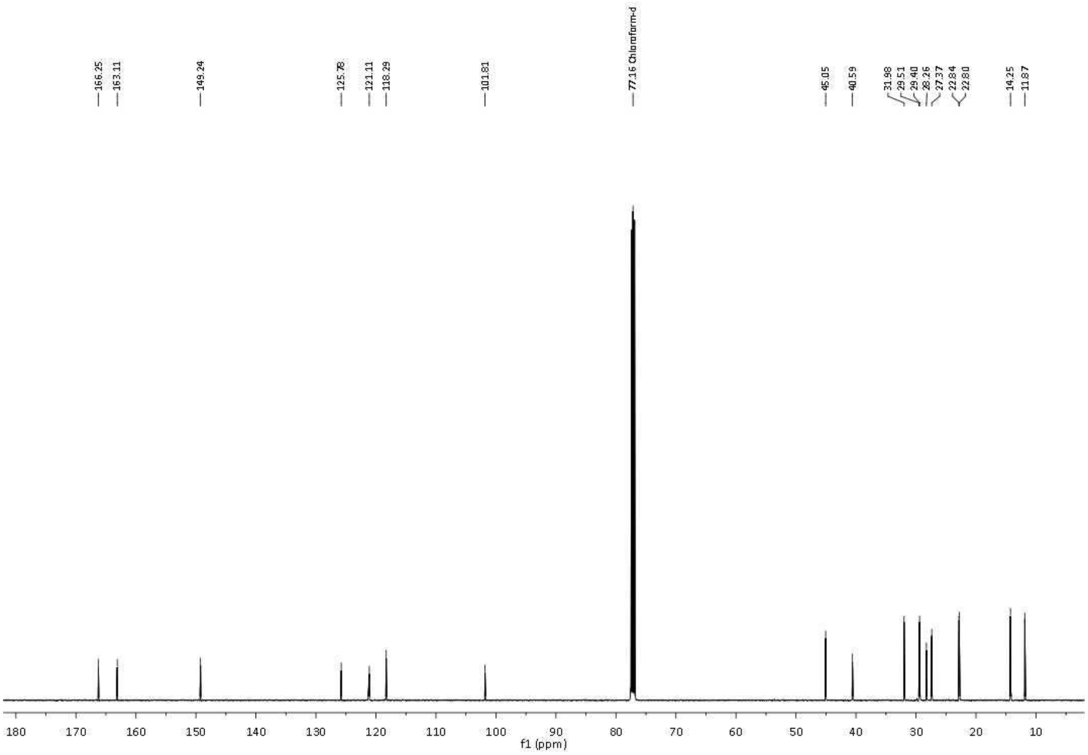

Figure S7. Image of ^{13}C NMR spectrum (126 MHz, $CDCl_3$).

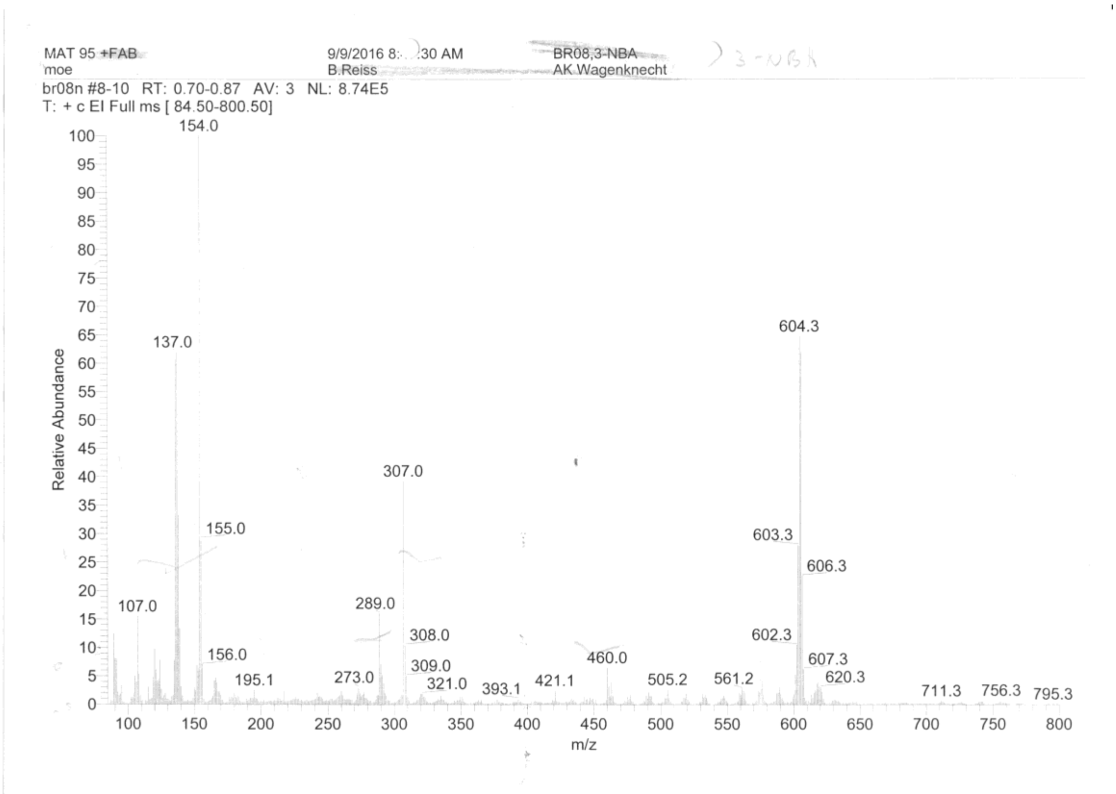

Figure S8. Image of mass spectrum (FAB).

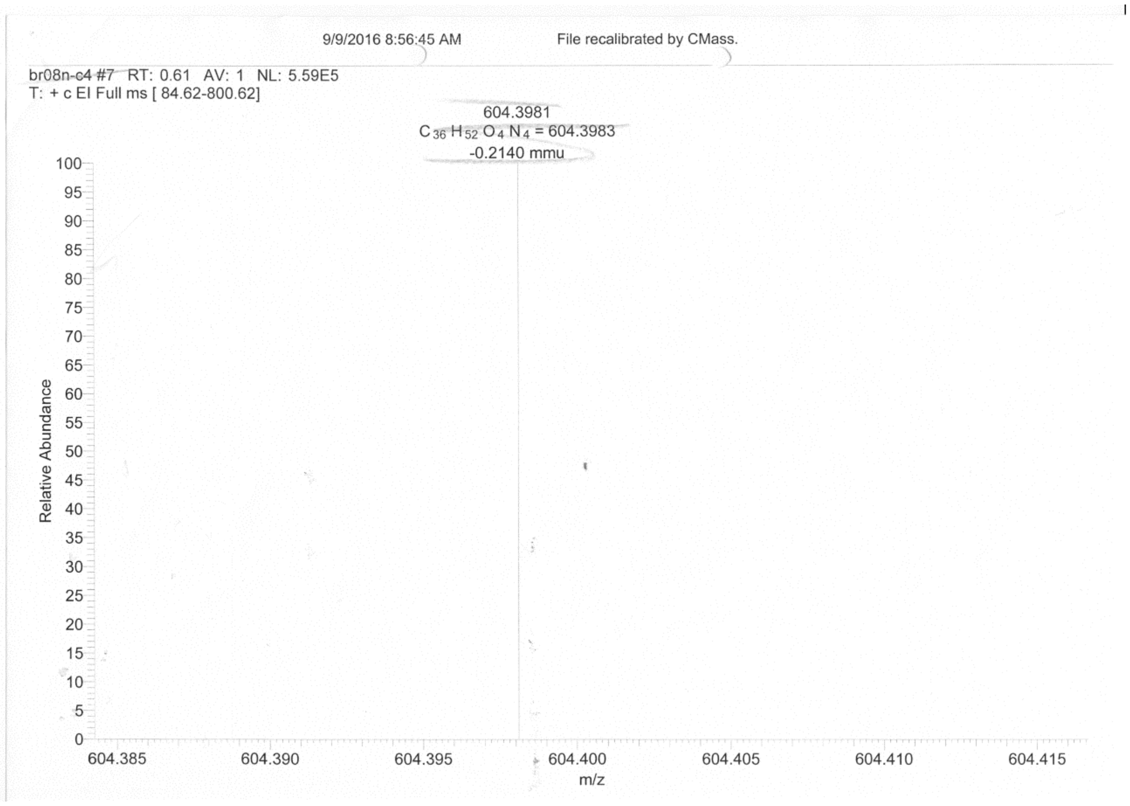

Figure S9. Image of HR mass spectrum (EI).

cNDI 3

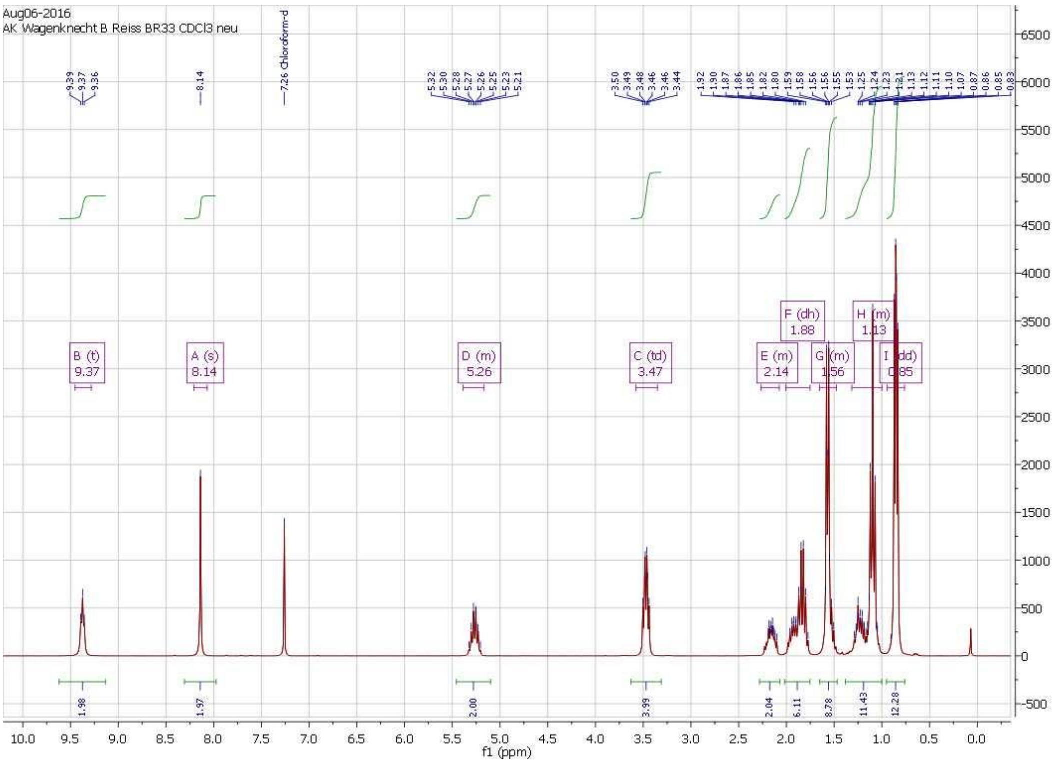

Figure S10. Image of ^{1}H NMR spectrum (500 MHz, $CDCl_3$).

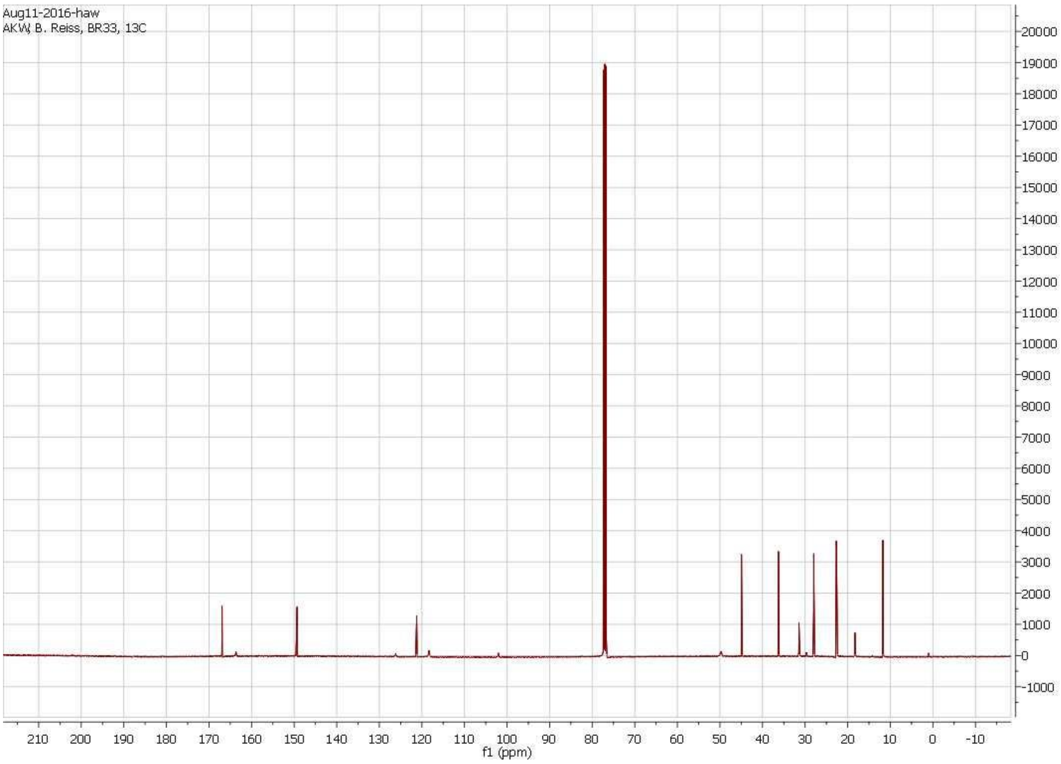

Figure S11. Image of ^{13}C NMR spectrum (126 MHz, $CDCl_3$).

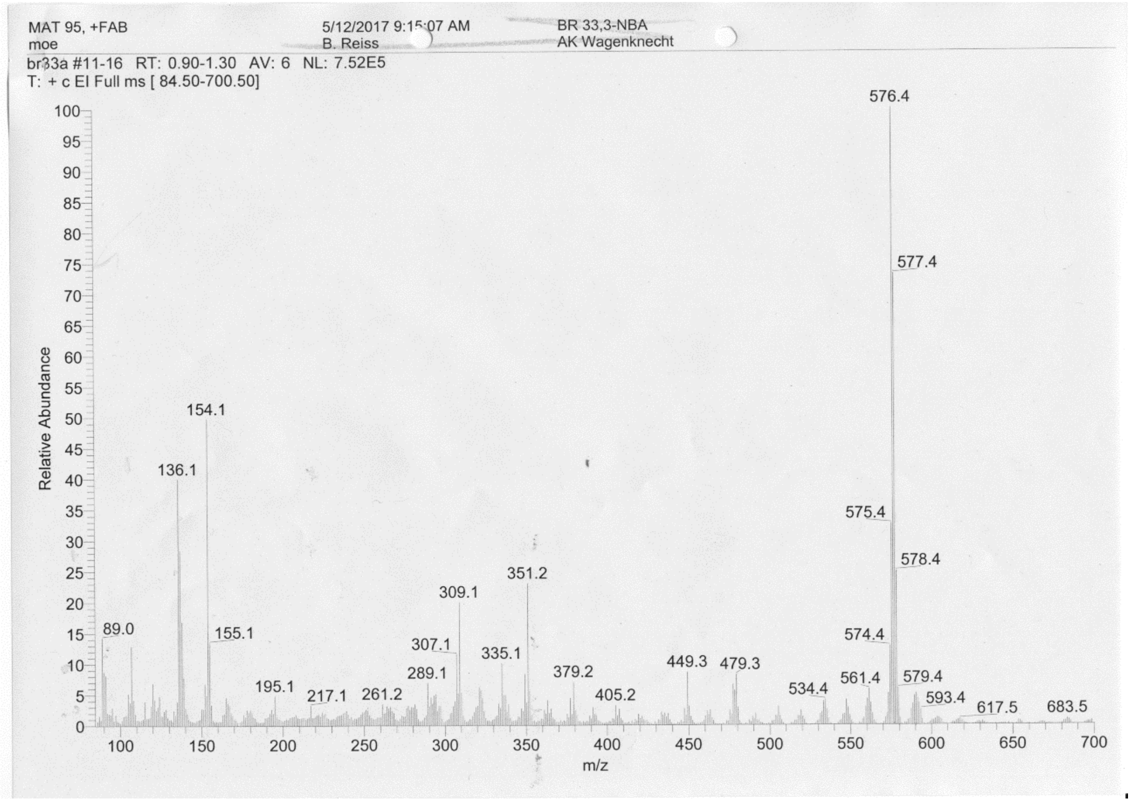

Figure S12. Image of mass spectrum (FAB).

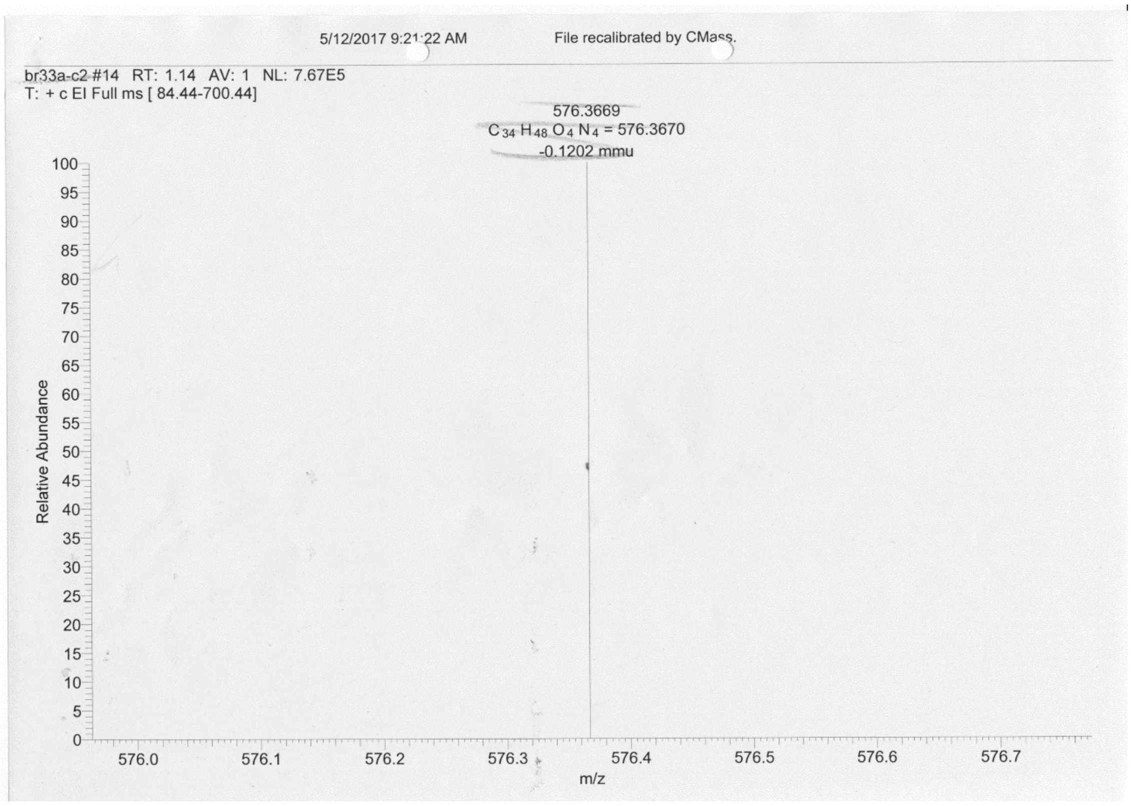

Figure S13. Image of HR mass spectrum (EI).

cNDI 4

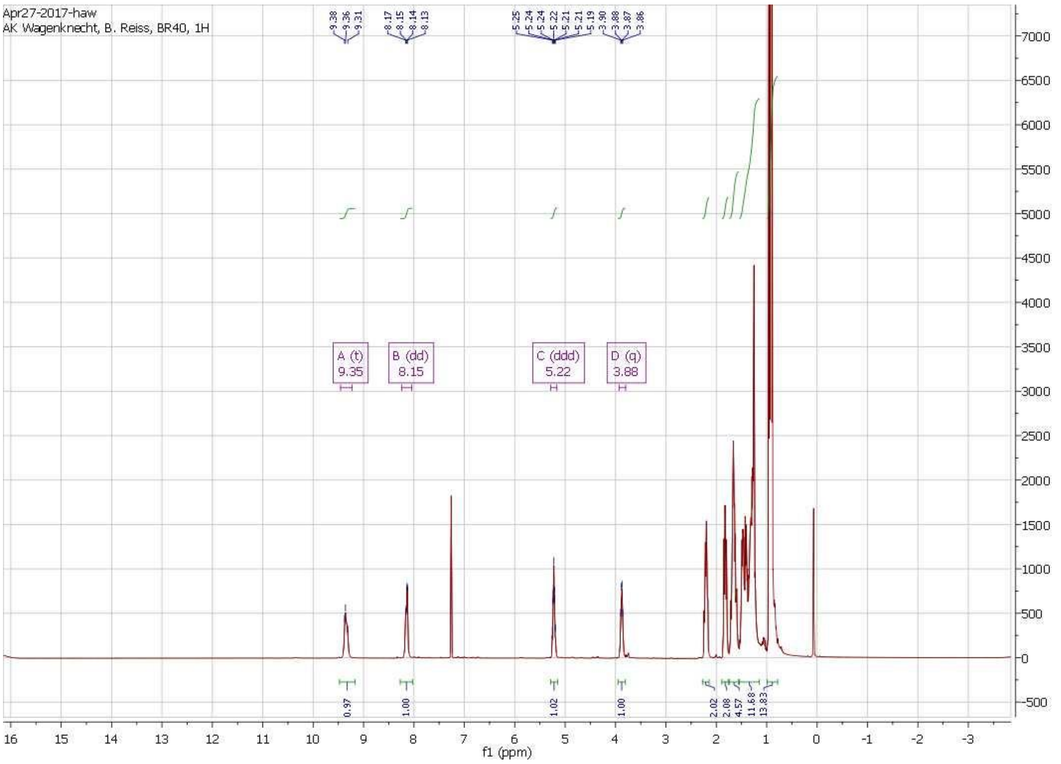

Figure S14. Image of ^{1}H NMR spectrum (500 MHz, $CDCl_3$).

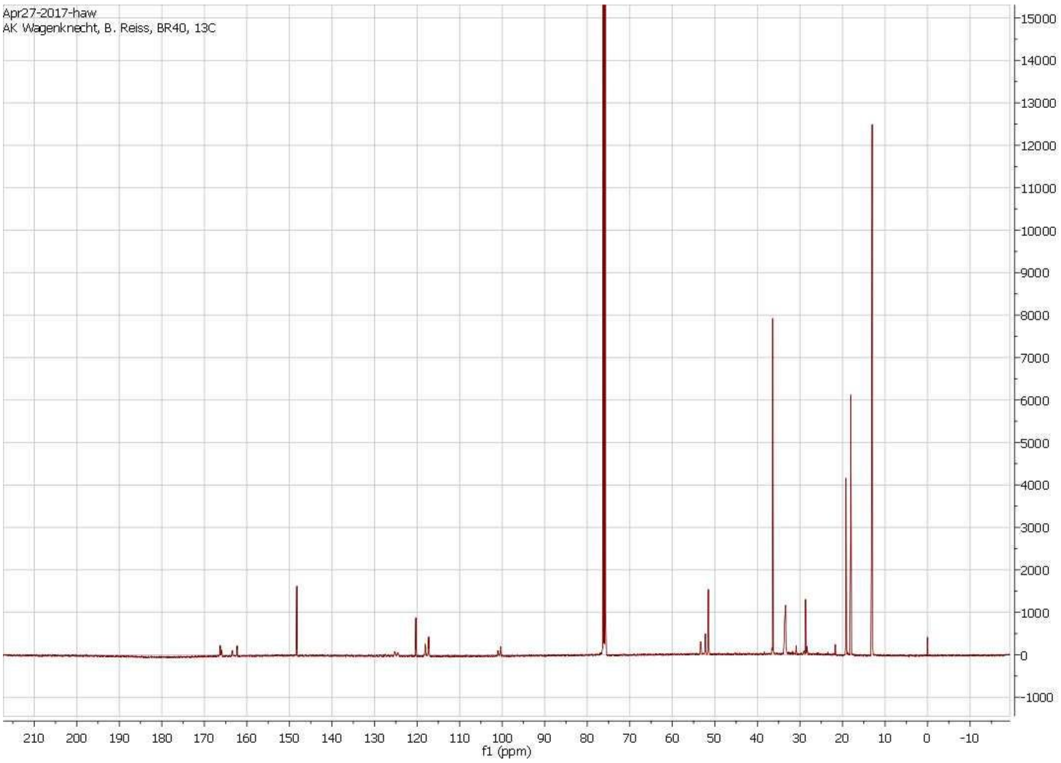

Figure S15. Image of ^{13}C NMR spectrum (126 MHz, $CDCl_3$).

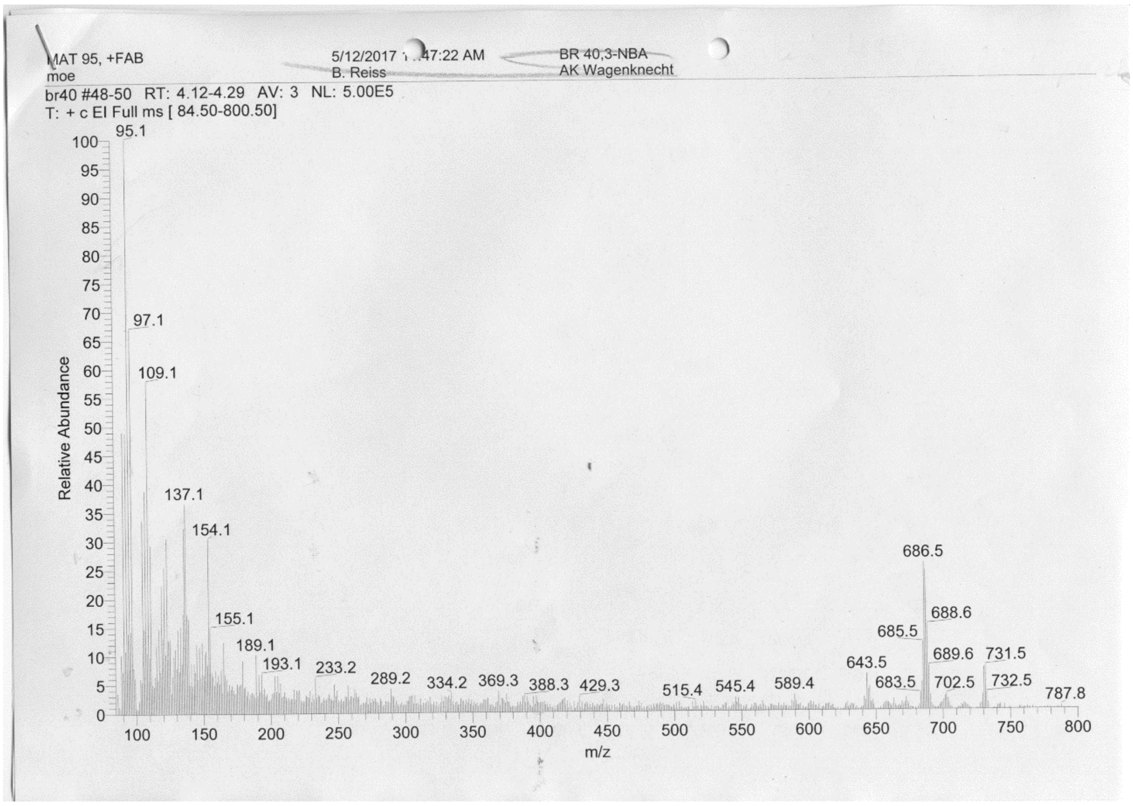

Figure S16. Image of mass spectrum (FAB).

cNDI 5

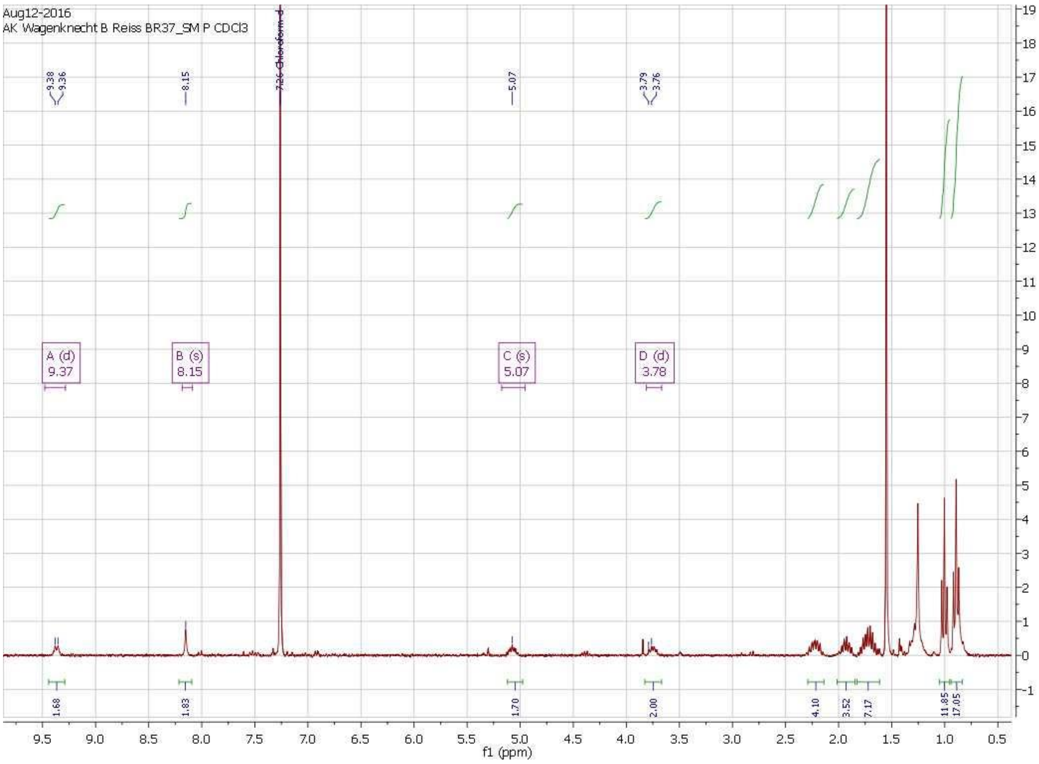

Figure S17. Image of ^{1}H NMR spectrum (500 MHz, $CDCl_3$).

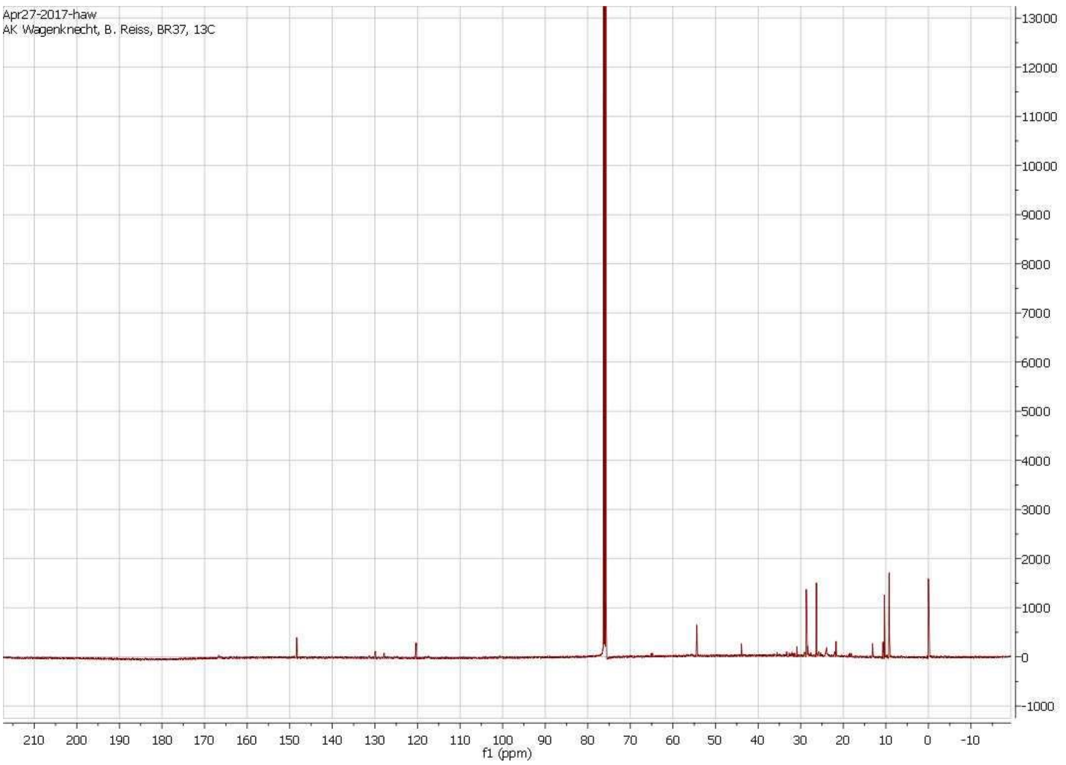

Figure S18. Image of ^{13}C NMR spectrum (126 MHz, $CDCl_3$).

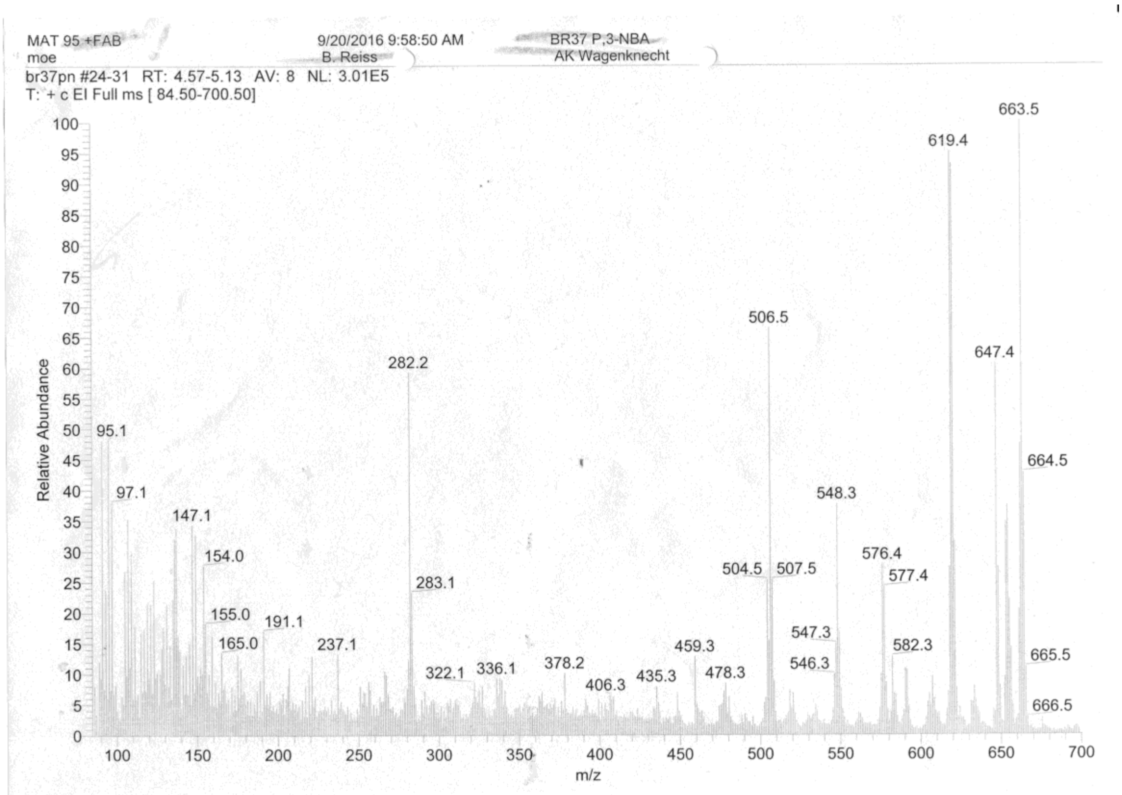

Figure S19. Image of mass spectrum (FAB).

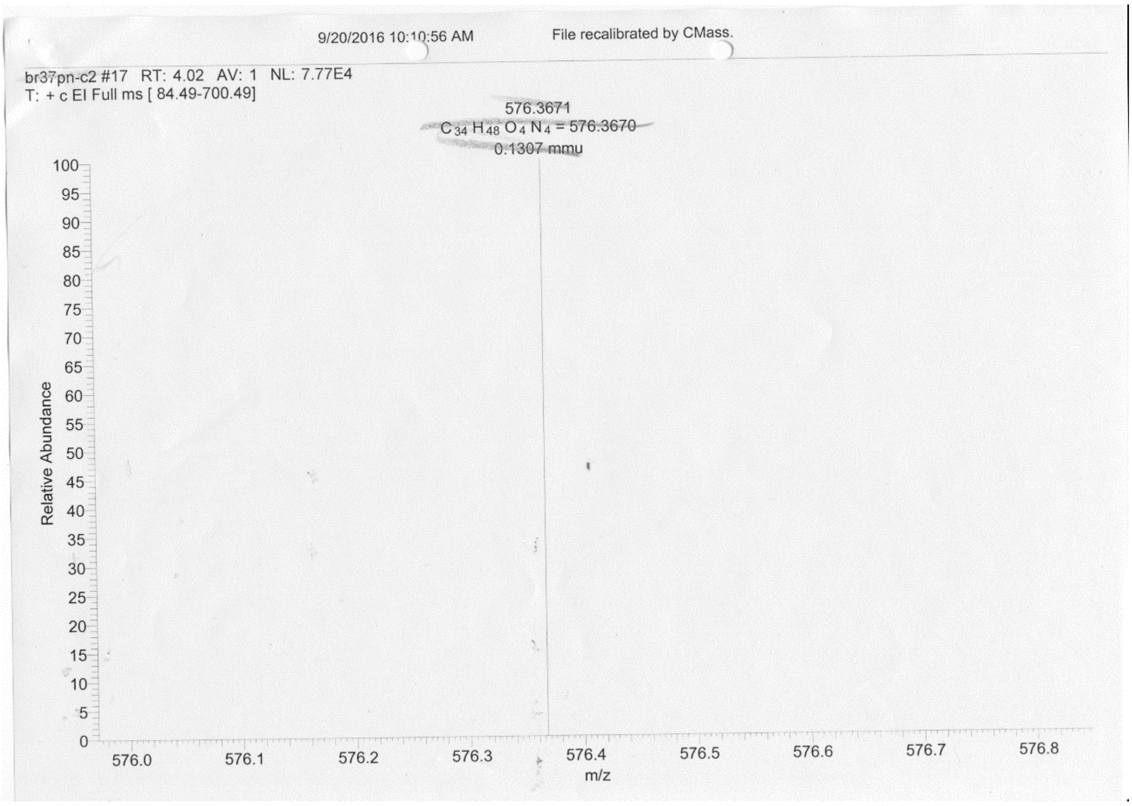

Figure S20. Image of HR mass spectrum (EI).

cNDI 6

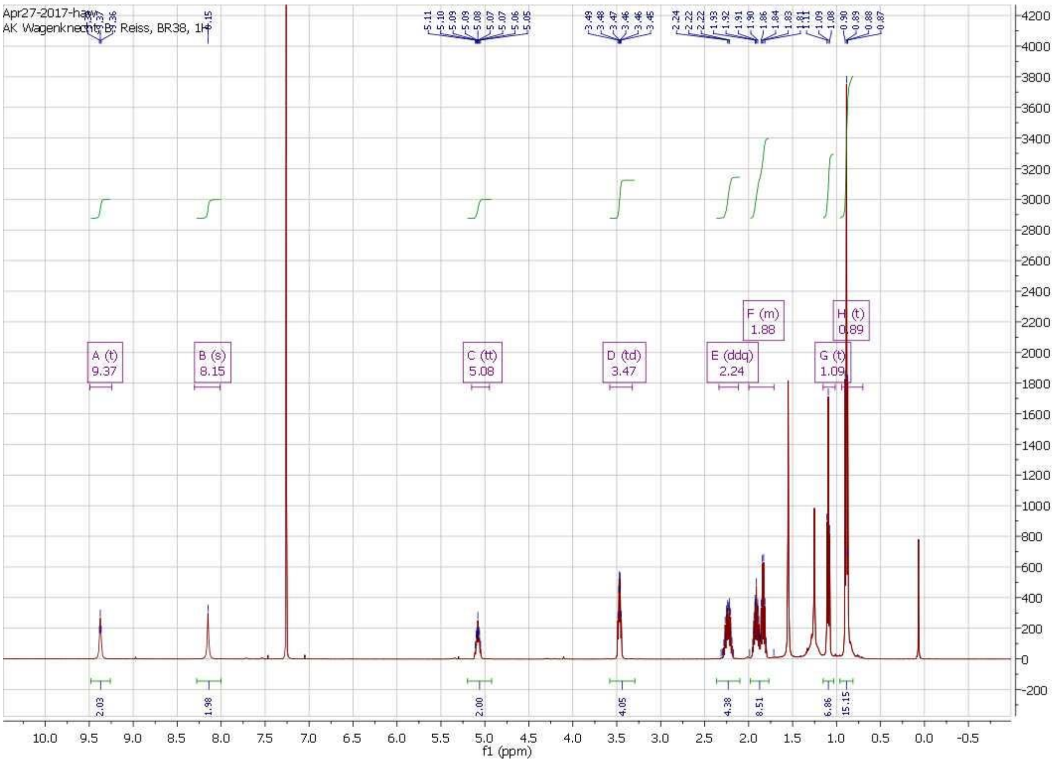

Figure S21. Image of ^1H NMR spectrum (500 MHz, $CDCl_3$).

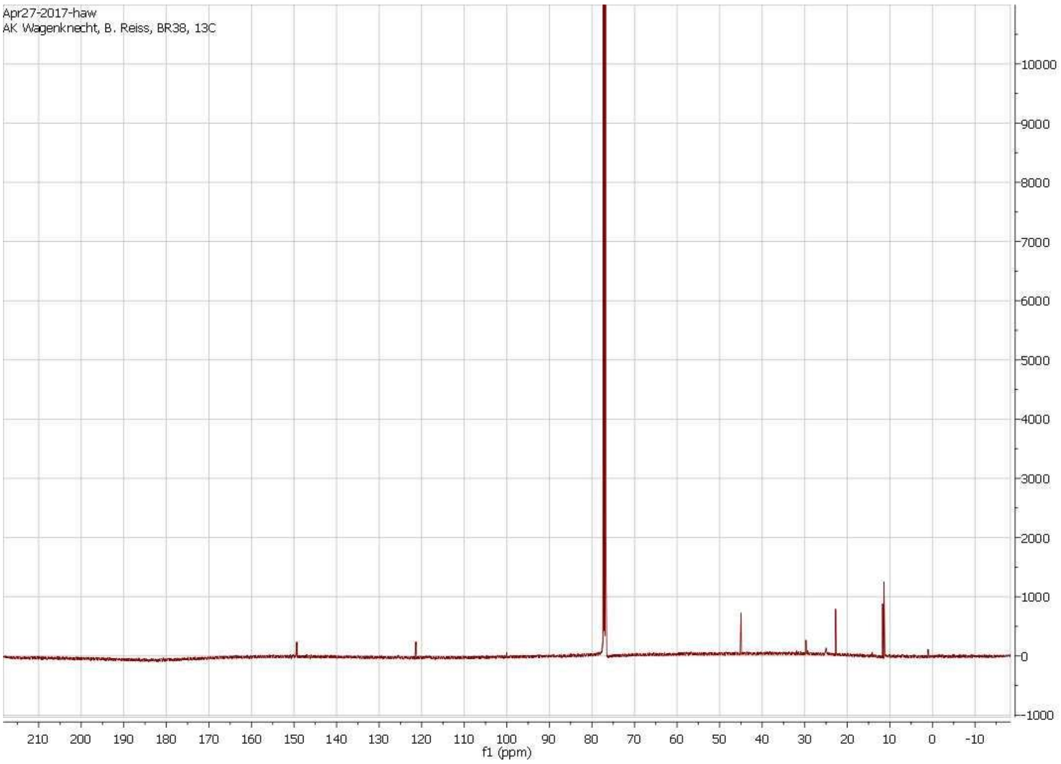

Figure S22. Image of ^{13}C NMR spectrum (126 MHz, $CDCl_3$).

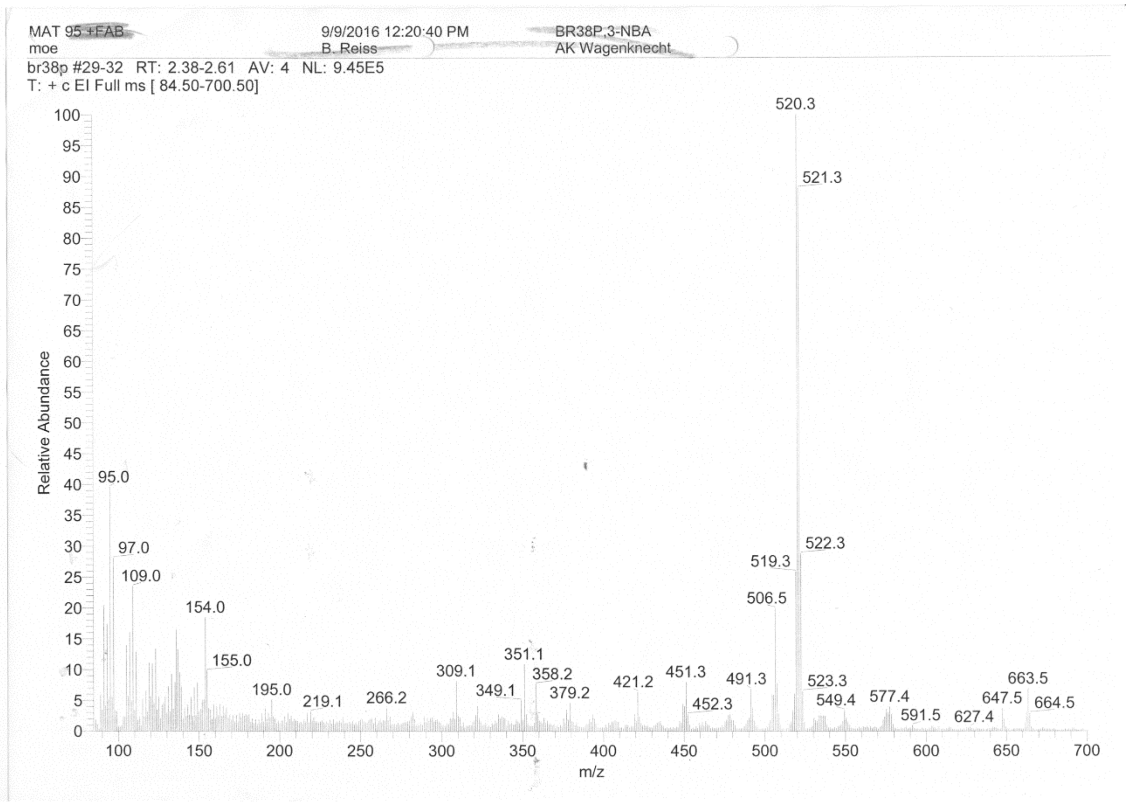

Figure S23. Image of mass spectrum (FAB).

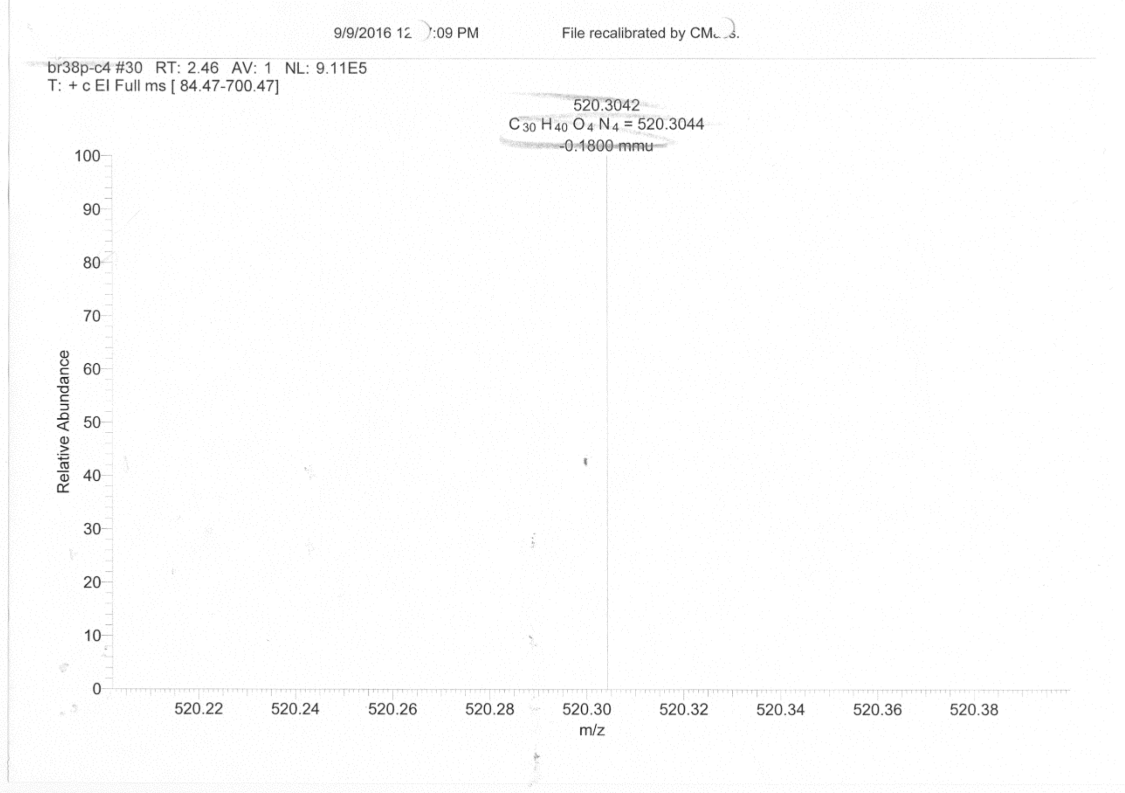

Figure S24. Image of HR mass spectrum (EI).

Photoredox catalytic experiments

Irradiation of the photochemical reaction was carried out using a setup which was designed and manufactured by the University of Regensburg and the workshop of the Institute for Physical Chemistry at KIT Karlsruhe (Figure S25). We warmly thank Dieter Waltz and Klaus Stree for their kind support with manufacturing the irradiation hardware. High-and low-power LEDs were used (Table S1). The photoreactions were irradiated from both sides. The temperature during the reaction time was controlled by cooling. For exact concentrations in the samples, see Tables and Figures in the main article. Organocatalyst **15** and the respective photoredox catalyst were dissolved. 1-Octanal (**12**), diethyl 2-bromomalonate (**13**), and 2,6-lutidine were added. The sample was degassed three times by the freeze-pump-thaw method. The

conversion of substrate **13** and the yield of product **14** were determined by ^{1}H NMR using the ratio of the singlets at 4.82 ppm (for **13**) and of the dublet at 3.73 ppm (for **14**). The debrominated diethyl malonate as side product has a characteristic singlet at 3.36 ppm.

The enantiomeric excess was determined after the following procedure (Scheme S1). After irradiation, the reaction mixtures was diluted with Et_2O and washed with water. The aqueous phase was extracted three times by Et_2O. The combined organic phases were washed by saturated aqueous solutions of 1) NH_4Cl, 2) $NaHCO_3$, 3) NaCl, dried over Na_2SO_4. The solvent was removed under vacuum. The crude product was purified by column chromatographie (SiO_2, hexane/Et_2O 25:2). The product **14** (20 mg, 69.8 µmol, 1.00 equiv) was dissolved in CH_2Cl_2 (1 mL). A mixture of (2*S*,4*S*)-(+)-pentanediol (>99% ee, 8.5 mg, 81.6 µmol, 1.17 equiv) and *p*-toluene sulfonic acid·H_2O 1.5 mg (7.88 µmol, 0.11 equiv) was added. The reaction was stirred at rt for 3 h. The solvent was removed under vacuum. The enantiomeric excess was determined by the ratio of the product integrals of the two dubletts at 3.70 ppm (acetale **16** from the (*S*)-enantiomer) and at 3.66 ppm (acetal **16** from the (*R*)-enantiomer).

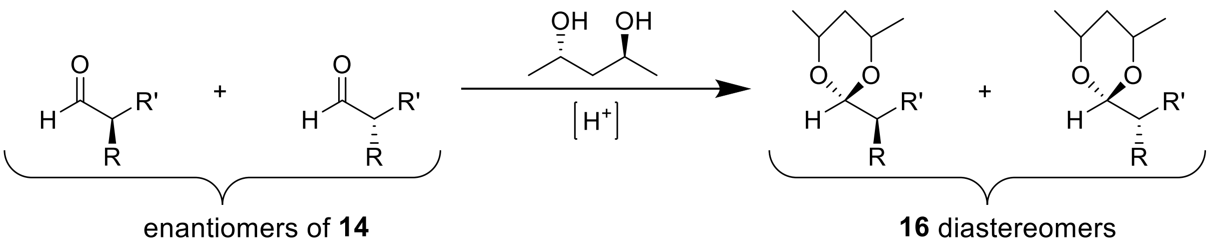

Scheme S1. Diastereomeric acetals **16** after reaction of product **14** with (2*S*,4*S*)-(+)-pentanediol for the determination of the enantiomeric excess.

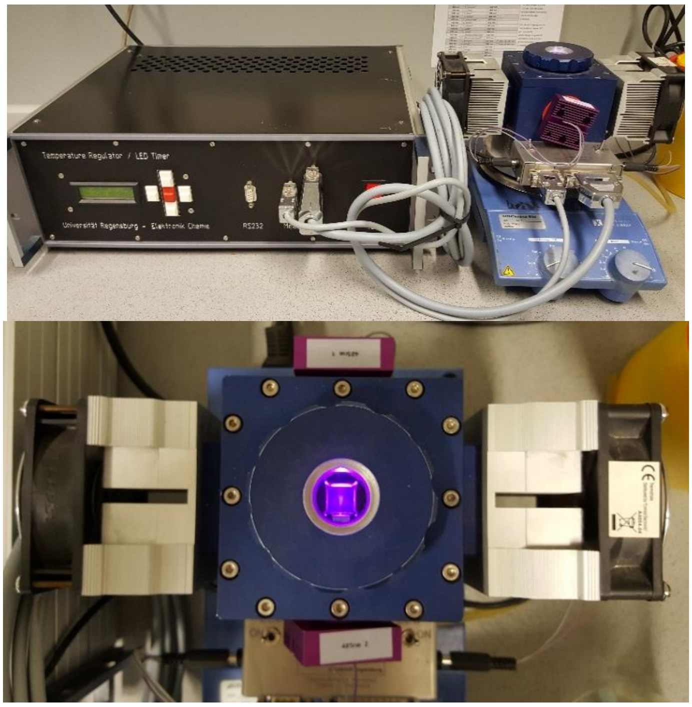

Figure S25. Images of the photoirradiation setup used for photoredox catalytic reactions

Table S1. Applied LEDs for irradiation.

Manufacturer	*Type*	$\lambda_{nominal}$ [nm]	λ_{max} [nm]	*Radiant power/ Current*
NICHIA	*UV NCSU034B*	385	**387**	540 mW (500 mA)
NICHIA	*NCSB119 32 lm*	470	**468**	32 lm (350 mA)
NICHIA	*NCSG119 130 lm*	530	**520**	130 lm (350 mA)
NICHIA	*NCSA119 80 lm*	590	**597**	80 lm (350 mA)
NICHIA	*NCSG119 53 lm*	620	**638**	53 lm (350 mA)
OSRAM	*Oslon SSL*	680	**683**	355 mW (350 mA)

Cyclic voltammetry

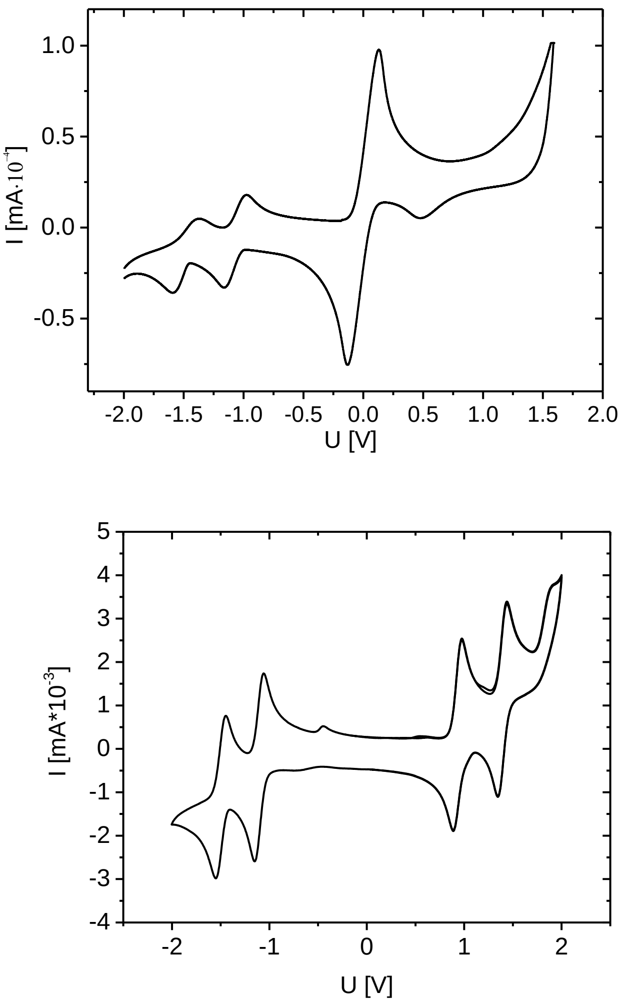

Figure S25. CVs of NDI **1** (top) and cNDI **2** (bottom) in CH_2Cl_2 (with 0.1 M NBu_4PF_6), scanning speed 100 mV/s. CVs were referenced by ferrocene and are given vs. SCE [S5].

References

S1. Hasharoni, K.; Levanon, H.; Greenfield, S. R.; Gosztola, D. J.; Svec, W. A.; Wasielewski. M. R. *J. Am. Chem. Soc.*, **1995**, 117, 8055-8056.

S2. Sasikumar, M.; Suseela, Y. V.; Govindaraju, T. Asian *J. Org. Chem.*, **2013**, *2*, 779-785.

S3. Graham, T. H.; Horning, B. D.; MacMillan, D. W. C. *Org. Synth.*, **2011**, *88*, 42-54.

S4. Chaignon, F.; Falkenstrom, M.; Karlsson, S.; Blart, E.; Odobel, F.; Hammarstrom, L. *Chem. Commun*., **2007**, 64-66.

S5. Pavlishchuk, V. V.; Addison, A. W. *Inorg. Chim. Acta*, **2000**, *298*, 97-102.
